# A Growth Mindset Scale for Young Children (GM-C): Development and validation among children from the United States and South Africa

**DOI:** 10.1371/journal.pone.0311205

**Published:** 2024-10-07

**Authors:** Melis Muradoglu, Tenelle Porter, Kali Trzesniewski, Andrei Cimpian

**Affiliations:** 1 Department of Psychology, Stanford University, Stanford, California, United States of America; 2 Department of Psychology, Rowan University, Glassboro, New Jersey, United States of America; 3 Department of Human Ecology, University of California, Davis, Davis, California, United States of America; 4 Department of Psychology, New York University, New York, New York, United States of America; National University of Lesotho, LESOTHO

## Abstract

Beliefs about the malleability of intellectual ability—mindsets—shape achievement. Recent evidence suggests that even young children hold such mindsets; yet, no reliable and valid instruments exist for measuring individual differences in young children’s mindsets. Given the potential relevance of mindsets to children’s achievement-related behavior and learning, we developed and tested the psychometric properties of the Growth Mindset Scale for Children (GM-C). Among other psychometric properties, we assessed this instrument’s (a) factor structure, (b) measurement invariance, (c) internal consistency, (d) temporal stability (test-retest reliability), (e) concurrent validity, and (f) cross-cultural robustness in samples of US children (Study 1; *N* = 220; ages 4 through 6; 50% girls; 39% White) and South African children (Study 2; predominantly grades 4 and 5; *N* = 331; 54% girls; 100% non-White). The GM-C scale exhibited four factors, representing beliefs about the instability of low ability, the malleability of low ability, the instability of high ability, and the malleability of high ability. The GM-C scale also demonstrated invariance across age, acceptable internal consistency (αs between .70 to .90), and moderate temporal stability over approximately one month (*r*s between .38 to .72). Concurrent validity was supported by significant relations between children’s scores on the subscales about low ability and their goal orientations (Studies 1 and 2), challenge-seeking behavior, and achievement in math and English (Study 2). These findings suggest that the GM-C scale is a promising tool for measuring mindsets in young children. We offer practical recommendations for using this new scale and discuss theoretical implications.

## Introduction

Students’ ability-related beliefs—that is, their beliefs about the nature of ability and its role in achievement outcomes—have downstream consequences for their motivation and achievement in school. One particularly important set of beliefs concerns whether intelligence can change. Prior work has shown that students who believe that intelligence is malleable (i.e., students with more of a “growth mindset”) do better in school than students who believe intelligence cannot be changed (i.e., students with more of a “fixed mindset”; [1, 2]): They are more likely to seek out academic challenges, show more persistence and adaptive behavior after experiencing failure, and thus learn more and have better achievement outcomes [3–5]. Despite the significance of students’ beliefs about the malleability of intelligence (i.e., their intelligence mindsets) for motivation and achievement in school, most mindset research to date has focused on adolescents and college students [3, 6, 7]. One obstacle to studying mindsets among preschool and elementary school students has been the lack of a measure of growth versus fixed mindsets suitable for young children. Given the potential relevance of these beliefs to children’s motivation and achievement in school, we developed and provide initial validation results for a scale that measures mindsets in young children (the Growth Mindset Scale for Children; GM-C).

### Mindsets about intelligence and their relation to motivation and achievement

Mindsets about intelligence consist of beliefs about the malleability of intelligence. These beliefs fall along a continuum from fixed to growth: Individuals with stronger fixed mindsets tend to view intelligence as a fixed, unchangeable entity, and individuals with stronger growth mindsets tend to view intelligence as a malleable quality ([8]; see also [9] for a recent conceptualization of mindsets as “fixed,” “growth,” and “mixed”, which contain elements of both fixed and growth beliefs). Importantly, mindsets about intelligence are a key element of an individual’s motivational framework—a system that (a) shapes behaviors prior to achievement events and (b) guides interpretations of and responses to achievement events [10, 11]. For instance, prior work has demonstrated that people’s mindsets influence their goals in achievement contexts [8, 12, 13]. Specifically, individuals with stronger fixed mindsets orient toward *performance goals*—their objective is to demonstrate their intelligence, and thus they prefer tasks that serve to validate their abilities. Individuals with stronger growth mindsets, in contrast, orient toward *learning goals*; they are concerned with increasing mastery of skill, and thus they prefer tasks that offer learning opportunities—even if these tasks are also more challenging and thus carry the risk of not performing well (for a review, see [14]; cf. [15]).

In addition to shaping the goals individuals select, mindsets guide individuals’ appraisals of and responses to achievement setbacks [3, 5, 14]. People with stronger fixed mindsets tend to regard failure as a sign of low ability, whereas people with stronger growth mindsets tend to regard failure as a sign that they may need to exert more effort or try a different strategy. To illustrate, in one study, seventh graders with fixed mindsets, compared to those with growth mindsets, were more likely to respond in helpless or defensive ways when confronted with a hypothetical failure scenario—they agreed with statements such as “I’m just not good at this subject” or “I didn’t really like the subject” [3; see also 16]. Similarly, individuals with stronger fixed mindsets have been shown to deploy other defensive behaviors—such as engaging in downward comparisons—to preserve their self-esteem [17]. People with stronger fixed mindsets are also less keen on opportunities to improve their abilities when they face failure [11]; they tend to report intentions to withdraw effort and engagement; and they sometimes even engage in academically dishonest behaviors, such as cheating [3]. In contrast, people with stronger growth mindsets are more likely to persist under the same circumstances, as well as engage in effort-based strategies [3] and take remedial courses of action that may enhance their abilities in the long term [11]. Taken together, this body of work suggests that students toward the fixed end of the mindset continuum are more likely to attribute their failures to low ability, and as a result they become demotivated by failure and respond to it in unproductive ways—by reducing engagement and effort. Individuals with stronger growth mindsets fare better when confronted with challenging events, in part because they react to such events with adaptive behaviors such as trying to identify the source of their mistakes, increasing effort, seeking help, or switching strategies.

Notably, because mindsets shape the activities students engage in and how they respond to achievement outcomes, mindsets can set students on different trajectories in school. That is, mindsets have been linked not only with different aspects of motivational frameworks (e.g., goals, attributions) but also with students’ actual *achievement*. For instance, among students who are facing academic risks, having stronger growth mindsets, or participating in an intervention that promotes growth mindsets, can lead to small but meaningful increases in achievement, including higher grades in school [3, 6, 18] and higher scores on standardized tests [19–21] (for reviews, see [22, 23]; cf. [24]).

### Mindsets in the preschool and elementary-school years

Mindsets about intelligence have attracted a significant amount of research attention, in part because of their consequences for student motivation and achievement. In spite of this level of interest, mindset research has largely overlooked the preschool and elementary-school years, focusing instead on middle-school-aged children (e.g., [3, 21]), high schoolers (e.g., [18, 19]), and college-aged students (e.g., [5, 17]) (for some exceptions, see [25–29]). In what follows, we discuss why the quantity of research on preschool and elementary school students’ mindsets has trailed the quantity of mindset research among older age groups (i.e., adolescents and adults).

In the 1970s and 1980s, research on young children’s achievement-related representations indirectly suggested that young children were incapable of holding mindsets [30–32]. More precisely, this body of research advanced the idea that children did not understand ability (as distinct from other relevant explanatory concepts such as effort) until they were around 10 years of age [30]. Some of the most influential evidence for this idea came from a series of studies in which children younger than 10 judged someone who had to work hard as smarter than someone who didn’t have to work as hard to achieve the same outcome [30]. Because young children did not seem to understand that someone who has more ability can succeed with less effort, it was assumed that they did not have a clear understanding of ability as distinct from effort.

The view that young children did not reason about performance outcomes in terms of ability, and thus could not hold beliefs about the nature and properties of ability, was reinforced by evidence on young children’s *behavior* in achievement contexts. In particular, young children seemed undaunted by failure experiences—they did not react in helpless or defeated ways when they made mistakes (e.g., [33]) and, even after repeatedly failing at a task, they tended to maintain high expectations of how they would perform in the future (e.g., [31]). This extraordinary optimism was seen as another illustration of young children’s “immature” reasoning about performance outcomes: Given that they (supposedly) understand such outcomes only in terms of effort, they also naturally assume that doing better in the future just requires trying harder.

Later research began to supplant the idea that young children (a) did not understand ability as a distinct causal factor that shapes performance outcomes and (b) are hopelessly optimistic in the face of failure. For instance, a growing body of evidence suggested that failure can cause negative responses even among preschool-age children, in the form of performance and persistence decrements and lowered self-evaluations (e.g., [34–38]; for a review, see [39]). Similarly, other studies demonstrated that children as young as 4 and 5 years of age are cognitively capable of reasoning about ability and effort as distinct concepts ([40]; see also [41]).

Despite this progress in understanding the structure of young children’s achievement representations, few attempts have been made to develop an instrument that is suitable for measuring young children’s mindsets. The instrument that is most widely used to measure mindsets in older populations [1, 2] consists of a set of eight statements that participants rate their agreement with on a 6-point Likert scale. Each of the items concerns general beliefs about the malleability of intelligence (e.g., “You have a certain amount of intelligence, and you really can’t do much to change it”). However, given the complex and abstract language in the items, the scale is only recommended “for children age 10 and older” ([1], p. 177).

### Existing measures of young children’s mindsets

Within the past decade, two scales have been developed to measure growth mindsets in young children. The first scale [25] has been used with children as young as 7 years of age. However, one limitation of this scale is that it includes aspects of motivational frameworks that are related to, but distinct from, mindsets about intelligence. For instance, this scale includes items concerning children’s challenge-seeking behavior (e.g., “How much would you like to do mazes that are very hard so you can learn more about doing mazes?”) and attributions (e.g., “Think of kids in your class who get a lot right on their schoolwork. Why do you think they get a lot right?”; see also [42]). In addition, the internal consistency of this scale is relatively low (α = .61), despite the fact that it contains as many as 18 items. The second scale [43] has been used with much younger children, namely 4- and 5-year-olds. In addition to its linguistic accessibility, another desirable property of the scale is that it comprises only three items and is therefore easy to administer (a) to very young children and (b) alongside other measures. However, one limitation of this scale is that it also includes aspects of children’s motivational frameworks that are distinct from mindsets. For instance, the scale asks about persistence behaviors (e.g., “If you got stuck building this castle, how hard would you keep trying?”). Further, the internal consistency of the scale was quite low as well (ω_o_ = 0.58).

From our perspective, the conflation of mindsets with mindset-related behaviors represents the most significant limitation of these two scales. However, two other limitations relevant to both scales are worth noting. First, the test-retest reliability of these scales was not examined. Second, these scales were constructed and validated using a single sample of children from a single city in the United States. Thus, the extent to which these measures (a) capture a stable, meaningful construct and (b) are usable across contexts is unclear.

In sum, a growing body of evidence supports the idea that young children do in fact hold mindsets about intelligence. At the same time, current tools to measure mindsets in children younger than 10 have significant limitations, including conflation of mindsets with other elements of motivational frameworks (e.g., goals), inadequate internal consistency, and a lack of evidence on test-retest reliability and cross-cultural robustness. Therefore, there is a need for valid and reliable measures of mindset that are brief and easy to use with young children. Such a measure can be used in research efforts aimed at understanding how mindsets emerge, change, and shape learning across childhood, as well as to inform interventions designed to foster growth mindsets.

### The developmental trajectory of young children’s mindsets

Although our central aim concerns the measurement of mindsets in young children, this work also addresses open theoretical questions on the developmental trajectory of children’s mindsets. Evidence from prior work on whether children’s mindsets become more growth-oriented or more fixed-oriented with age, or are developmentally stable instead, offers a mixed picture. Some work points to stronger fixed mindsets among *younger* children. For instance, in one study with fourth through sixth graders, older children viewed ability as more controllable than younger children did [44]. Similarly, another study with third through sixth graders found that older children endorsed fixed views of ability less strongly than younger children did ([13]; see also [27, 40]). Other work, however, has pointed to stronger fixed mindsets among *older* children. For example, 11- to 13-year-olds viewed ability as less malleable than 7- and 8-year-olds did [45]; other work has documented similar age trajectories among second through fifth graders [46] and among fourth through sixth graders [44]. Further complicating this picture, at least two studies have found no age-related change in children’s beliefs about intellectual ability: one that investigated second through fifth graders’ beliefs about ability [46] and another that compared kindergartners’, second graders’, and fourth graders’ beliefs [47]. These inconsistent results might stem from differences in measurement. Without a reliable, validated measure of young children’s mindsets, researchers have to devise their own measures, and differences in measurement can lead to inconsistent findings. By developing and validating a measure of mindsets suitable for use with young children, we hope to bring some clarity to this complex empirical landscape.

In addition to understanding how children’s mindsets change with age, it is important to understand how well children’s mindsets cohere with the other elements of their motivational frameworks and with their achievement across development. Currently, this issue is relatively underexplored, and the findings (sparse as they are) are again not entirely consistent. In one relevant study [47], fixed mindsets were correlated with performance goals for fourth graders, but not kindergartners or second graders. However, another study found that mindsets (measured alongside other elements of children’s motivational frameworks; see discussion above) predicted math achievement longitudinally among first and second graders [48]. In contrast, a third relevant study found that mindsets were not correlated with learning goals in a sample of first through eight graders, and the magnitude of this (non)relation did not change significantly across grades [49]. In summary, although one might expect that—as children progress through preschool and elementary school—their mindsets become more tightly linked with the rest of their motivational frameworks and with their achievement, the evidence so far does not support this simple developmental narrative. Our scale development effort—which involved measures tapping a broad range of elements of children’s motivational frameworks included for validation purposes—presents an opportunity to shed additional light on the cohesiveness of children’s motivational frameworks across development, and paves the way for more focused study of this matter in the future.

### The present research

The goal of the present work was to develop a mindset measure that (a) contained items that were linguistically and conceptually accessible for children as young as 4 years of age, and (b) assessed children’s beliefs about the malleability of intellectual ability (as distinct from other motivationally relevant beliefs and behaviors) precisely, reliably, and validly.

To evaluate the internal structure of this new measure, the Growth Mindset Scale for Children (GM-C), we used exploratory factor analyses (EFAs) and confirmatory factor analyses (CFAs) with separate samples of children. To determine whether the GM-C is interpreted similarly by children of different ages, we tested measurement invariance using multi-group CFAs. Establishing measurement invariance allowed us to then ask whether children’s mindsets differed by age (e.g., [48]), as well as whether they related differently to other components of children’s motivational frameworks as a function of age. To assess the internal consistency of the items and the test-retest reliability of the scale, we used Cronbach’s alpha and zero-order Pearson correlations, respectively.

To evaluate the GM-C scale’s validity, as well as investigate questions about the coherence of children’s motivational frameworks across development, we administered a number of measures that typically relate to mindsets in older populations. Perhaps most importantly, we measured children’s learning versus performance goals (Studies 1 and 2), which are theorized to flow directly from children’s growth versus fixed mindsets, respectively (e.g., [8]). In addition, prior work has found that individuals with stronger growth (vs. fixed) mindsets tend to engage in more challenge-seeking behaviors (e.g., [3, 16]), show more perseverance and less negative reactions after experiencing failure (e.g., [4, 5]), and earn better grades in school (e.g., [3, 6]; see also [21, 28]). Therefore, we also measured children’s preference for challenges (Study 2), their persistence (Study 1), their affective response to failure (Study 1), and their achievement in two subjects (math and English; Study 2).

Finally, we recruited a Western sample (from the US, Study 1) and a sample from the Global South (specifically, from South Africa, Study 2) to assess the cross-cultural robustness of the scale. Often due to a lack of sample diversity during the scale development process, the generalizability of new instruments to other cultural contexts tends to be low [50]. This is largely the same limitation that psychological research suffers from more generally, especially given the extent to which research in the field is dominated by samples from WEIRD (Western, Educated, Industrialized, Rich, and Democratic) countries [51]. The inclusion of participants from multiple cultures in the scale development process is a notable improvement over the typical procedure for scale development, which involves construction and assessment of scale performance among Western participants, with later adaptation or use among other samples. Table 1 provides an overview of the studies’ aims and statistical approaches.

**Table 1:** Overview of research.

	Study aims	Statistical approach	Time of data collection	Sample source and demographics
Study 1 (*N* = 220)	Assess dimensionality (factor structure) of the GM-C	Exploratory factor analysis (EFA) (sample 1); confirmatory factor analysis (CFA) (sample 2)	July 2017-March 2018 (Sample 1); April 2018-February 2019 (Sample 2)	Four- to six-year-old US American children in a large, urban area in the Mid-Atlantic US
	Test concurrent validity of the GM-C	Regression models with GM-C subscale scores predicting children’s (1) preference for learning goals, (2) upward comparisons, (3) persistence, and (4) affective response to failure; zero-order Pearson correlations between GM-C subscale scores and children’s open-ended justifications		
	Assess internal consistency of GM-C	Cronbach’s alpha		
	Test-retest reliability of the GM-C	Zero-order Pearson correlations between subscale scores at Time 1 and Time 2		
	Test whether the GM-C is invariant across age groups (4-, 5-, and 6-year-olds)	Multi-group CFAs		
Study 2 (*N* = 331)	Assess internal consistency of GM-C	Cronbach’s alpha	April & May 2018	Predominantly fourth- and fifth-grade Xhosa- and Afrikaans-speaking children in the Western Cape, South Africa
	Test concurrent validity of the GM-C	Regression models with GM-C subscale scores predicting children’s (1) preference for learning goals, (2) challenge-seeking, (3) math achievement, and (4) English achievement		
	Test convergent validity of the GM-C	Zero-order Pearson correlations between GM-C subscale scores and Theory of Intelligence scale		

## Study 1

### Method

Studies 1 and 2 were approved by the Institutional Review Boards at New York University (IRB # FY2016-1163 “How do children make sense of the world?”) and UC Davis (IRB # 1872614–1 “Measuring growth mindset”), respectively. Parents provided written consent on behalf of their children.

#### Approach to designing a measure of young children’s mindsets

Our goal for the new scale was to capture young children’s beliefs about the malleability of intellectual ability with a high degree of precision and in a way that was linguistically and conceptually accessible to this participant population. In contrast to prior measurement efforts that mixed multiple aspects of children’s motivational frameworks [25, 43], we homed in on children’s *beliefs* about ability (exclusive of other aspects of their motivational frameworks), with several theoretical and practical considerations—detailed next—guiding our approach to developing the items in our scale.

The first obstacle to constructing a scale of mindsets for young children is their limited linguistic abilities. Age of acquisition norms suggest that words such as “intelligence” and “intelligent” are not reliably understood by English-speaking children until about age 8 or 9 [52]. Although simpler words exist that are similar in meaning (e.g., “smart”), there are important ambiguities in the meaning denoted by both “intelligent” and “smart.” For example, these words can be used to describe someone who is knowledgeable (“crystallized intelligence”) or, alternatively, someone who is quick-witted (“fluid intelligence”), regardless of how much they know. Cognitive interviews suggest that even adults are sometimes unsure how to interpret the items in the classic mindset scale for this reason [53, 54]. Thus, switching from “intelligent” to “smart” would not resolve the deeper semantic issue here, which pertains to the ambiguity in the shared meaning of these words. For this reason, we decided to ask children about ability in specific intellectual domains that would be familiar to 4- to 6-year-olds rather than asking them about intellectual ability more generally. We reasoned that the shared variance across items pertaining to specific domains could serve as a proxy for children’s beliefs about domain-general intellectual ability, especially given evidence that domain-specific beliefs are indicative of global mindset beliefs [55]. Notably, this domain-specific measurement strategy is becoming increasingly common in mindset research (e.g., [28, 55]).

Evidence that young children distinguish between ability in different domains is fairly limited and mixed. For instance, in a study with third- to sixth-grade children, children’s beliefs about the stability of ability did not vary by domain (math vs. social science; [13]). In a more recent study, however, even first graders regarded high intellectual ability as more central for success in math than in reading or writing ([56]; see also [26]). Based on a review of the literature, we decided to assess children’s mindsets about the domains of math, spelling, and drawing, which satisfied four key criteria: They (a) are familiar to this young age group, (b) are generally perceived as intellectual or cognitive (as opposed to physical, such as sports), (c) elicit variability of opinion among adults regarding the fixedness versus malleability of ability (e.g., [1, 2]), and (d) are different enough from each other (content-wise) that the shared variance among them could approximate children’s beliefs about intellectual ability more broadly conceived.

After deciding on the three domains, our next goal was to identify what to ask young children in order to adequately capture their thoughts about whether intellectual ability can change. Beliefs about the *changeability* of ability are also complex: They can involve assumptions about the origins of ability (innate vs. acquired), its stability or constancy across time and/or contexts, and the degree to which it can be shaped by effort or other interventions. Notably, prior work has consistently demonstrated that these nuances are empirically distinguishable—even among young children—and, perhaps surprisingly, sometimes only weakly related to one another [13, 44, 46]. In light of these considerations, and consistent with recent calls for more multifaceted measures of mindsets that can better capture the complexity of people’s reasoning about ability [57], we assessed *two* perspectives on changeability that we perceived to be conceptually central to mindsets: children’s beliefs about the extent to which ability is (a) stable across time and (b) responsive to intervention. To make the questions as concrete and easy to follow as possible, we introduced participants to a drawing of a child character, provided the character with a name, and said that the character currently was either good or not good in one of the three domains. Once the stage was set in this way, we simply asked children whether the character’s ability would ever change: “Will it always be this way?” This *instability* question taps the core of the mindset construct and has the advantage of being very simple, but it is also somewhat vague, in that it does not specify the character’s future circumstances: It may be hard for children to know if the character’s ability will change if they do not know what the character will do in the future or what will happen to them. The next, *malleability*-focused question was more concrete, and it also allowed us to tap a distinct aspect of children’s mindsets. Specifically, we asked children to consider whether—and to what degree—a specific change in the character’s environment would precipitate a change in their ability. In particular, we described the character as moving to a new environment that provided them with (or deprived them of) opportunities to practice their skill in the relevant domain. We then asked children whether the character was good or not good in that domain after being in their new environment for a while. In formulating this malleability question, we avoided wording that suggested *self-initiated* attempts to withhold or exert effort (e.g., “she decided to work very hard”), which we suspected might prompt socially desirable answers (e.g., “yes, she is good now”). Instead, we described a change in the character’s *environment* (specifically, moving to a different school).

As already mentioned, another distinctive aspect of our measure was that we varied the initial ability level of the characters in the vignettes. Prior work has shown that children’s judgments about the changeability of intellectual ability depend on its initial level, with children viewing high ability as less changeable than low ability [45, 58]. Considering these findings, we deemed it important to vary the initial ability level of the characters in the vignettes (see also [57]). Doing so also allowed us to make the content of the questions even more concrete, which is desirable given that the scale is intended for young children.

#### Participants

To assess the dimensionality of the scale in Study 1, we recruited one sample of participants for the EFA and a second sample for the CFA. Participants in Sample 1 were 112 4- to 6-year-old children (56 girls, *M*_age_ = 5.50 years, *SD* = 0.83 years, range = 4.08 to 6.94) recruited from a large city in the Mid-Atlantic US between July 2017 and March 2018. For factor analytic purposes, a sample of 5–10 participants per item (up to 300 participants) is generally recommended [59]. Because our scale comprised 12 items, the sample sizes here (*N* = 112 and *N* = 108) fall within the desirable range of 60–120 participants. There was a roughly equal number of children per age group (*n* = 37 4-year-olds, *n* = 38 5-year-olds, and *n* = 37 6-year-olds). Children were tested either in a museum (*n* = 64) or in a university laboratory (*n* = 48). Of the children whose parents reported their child’s race and ethnicity (95%), 49% were Non-Hispanic White or Caucasian, 21% were Multiracial, 14% were Asian or Pacific Islander, 8% were Hispanic/Latinx, 4% were Black or African American, and 4% belonged to other groups. The median household income was $200,000; 38% of parents did not report this information. Exclusion criteria were predetermined: (1) children who are not between the ages of 4 and 6 (inclusive), (2) children who opt to end the study early, (3) caregivers who opt to end the study early, and (4) caregivers who unduly influence their child’s response or otherwise interfere during the session. Seventeen additional children were tested but excluded because they or their parent opted to end the study early (*n* = 15) or because they were out of the target age range (*n* = 2).

Participants in Sample 2 were 108 4- to 6-year-old children (54 girls, *M*_age_ = 5.53 years, *SD* = 0.78 years, range = 4.06 to 6.91) recruited from a large city in the Mid-Atlantic US between April 2018 and February 2019. There was a roughly equal number of children per age group (*n* = 37 4-year-olds, *n* = 35 5-year-olds, and *n* = 36 6-year-olds). Children were tested either in a quiet room at their school (*n* = 82) or in a university laboratory (*n* = 26). Of the children whose parents reported their child’s race and ethnicity (89%), 29% were Non-Hispanic White or Caucasian, 24% were Multiracial, 21% were Hispanic/Latinx, 14% were Asian or Pacific Islander, 5% were Black or African American, and 8% belonged to other racial-ethnic categories. The median household income was $150,000; 83% of parents did not report this information. Although few parents self-reported socioeconomic status information, we note that 41% of the sample was recruited from an elementary school in which most students (61%) were eligible for free or reduced-price lunch (indicating that their families had low incomes). Six additional children were tested but excluded because they opted to end the study early or refused to answer questions.

Combining the two samples resulted in a sample size of *N* = 220. A sensitivity analysis assuming an α level of .05 (one-tailed) indicated that this sample size had 80% power to detect effects considered “small” by convention (*r* = .17; [60]).

#### Materials and procedure

*The GM-C measure*. The full measure is available on the Open Science Framework (OSF): https://osf.io/hm2yg/?view_only=7da7b40cd6724fae84f1ed3b493f79b7. As stated previously, we constructed vignettes about math, spelling, and drawing. Three vignettes concerned characters that were unskilled in a domain (*low-ability vignettes*); the other three vignettes concerned characters that were skilled in a domain (*high-ability vignettes*). Four of the characters were girls, and two of the characters were boys. Character gender within a domain was consistent: The two math vignettes involved girl characters, as did the two drawing vignettes; the two spelling vignettes involved boy characters. To ensure that ability level (i.e., high or low) was the only element that varied between vignettes about a given domain, we deliberately designed the vignettes to have the gender of the characters constant within a domain. Doing so necessarily prevented us from having an equal number of girl and boy characters in the vignettes. In other words, having an equal number of girls and boys in the vignettes would necessarily require gender to vary *within* a domain, which we deemed undesirable for the reasons articulated above. To ensure that children encoded the ability information from the vignette, the researcher administered a memory check after introducing the character. If children answered incorrectly, the researcher corrected the child and continued administering the study. For each of the six vignettes, children were asked two questions (always administered in the order presented below), for a total of 12 items. Responses were scored on a scale from 0 to 1, with higher numbers reflecting a stronger growth mindset.

#### Instability of ability

To assess children’s beliefs about the *instability* of the character’s ability, the researcher asked the following question: e.g., “Will it always be this way? Will Jamie always be not-very-good at math?” (see S1 Table in S2 File [SOM] for additional examples). The researcher then asked the child how confident they were (“How sure are you about this? Are you sort of sure? Or really sure?”), which yielded four possible responses (yes *and* really sure = 0, yes *and* sort of sure = 0.33, no *and* sort of sure = 0.67, no *and* really sure = 1).

#### Malleability of ability

To assess children’s beliefs about the malleability of the character’s ability, the researcher first read the child a short vignette in which the character was described either as receiving (low-ability vignettes) or as not receiving (high-ability vignettes) the opportunity to practice. For example, in the low-ability vignette about math, children heard:

Now let me tell you what happened with Jamie. When Jamie was a little older, she moved to a school far away. At this school, kids do a lot of math. After Jamie started at this far-away school, she got to practice math a lot. Jamie did a lot of math at this school.

The researcher then asked the child whether they thought there was a subsequent change in the character’s abilities: e.g., “Jamie was at this school for a long time. When she left this school, was she good at math or not good at math?” The researcher followed up with a three-point scale, accompanied by three smiley or frowny faces of increasing intensity (“Was [s]he sort of good/not good, good/not good, or really good/not good?”). This yielded six possible responses (*really not good* = 0, *not good* = 0.2, *sort of not good* = 0.4, *sort of good* = 0.6, *good* = 0.8, *really good* = 1.0). Responses to this item were reverse-scored for the high-ability vignettes, which concerned characters who started out skilled in a domain.

GM-C was always administered first in study sessions. The vignettes within the measure were presented in one of twelve orders (Sample 1) or in one of six orders (Sample 2). The three high-ability vignettes were always presented together as a block, and the same was true of the three low-ability vignettes.

*Validation measures*. We investigated the validity of the GM-C measure by assessing how it relates to theoretically relevant cognitions, attitudes, and behaviors. Based on prior work, we expected that children who were more growth-oriented, as measured by the GM-C scale, would be more likely to orient toward learning (rather than performance) goals, engage in upward (rather than downward) comparisons, and show more persistence and less negative affect after failure. To keep the sessions to a reasonable length and avoid taxing children’s attention spans, we distributed these validation measures across the two samples: Some measures were exclusively administered to Sample 1, while others were exclusive to Sample 2. The only exception was the measure of learning versus performance goals, which we administered in both samples due to its theoretical significance as a direct outcome of mindsets. See the SOM for response distributions of these measures in Studies 1 and 2 (S4–S7 Figs in S2 File).

#### Learning versus performance goals (Samples 1 and 2)

In the goals task (adapted from [12]), the researcher presented the child with two opaque boxes and stated that one of the boxes contained puzzles that the child would learn a lot from but might sometimes make mistakes on (learning goal), and that the other box contained puzzles that were easy, so the child would do well (performance goal). The researcher then asked the child to point to the box of puzzles they preferred to play with (*easy box* = 0, *challenging box* = 1).

Upward versus downward comparisons (Sample 1): The upward versus downward comparison measure resembled an “I Spy” game: The researcher asked the participating child to circle as many target objects (here, buses) as they could find in a laminated picture that depicted dozens of objects. The researcher told the child that they “only had a little bit of time” to find the buses. However, to keep performance consistent across children, the researcher always stopped the child and announced that time was up after the child had circled nine buses. They then asked the child whether they wanted to look at the work of a peer who performed better than they did (i.e., found more buses; upward comparison) or at the work of a peer who performed worse than they did (i.e., found fewer buses; downward comparison) (*fewer buses* = 0, *more buses* = 1).

#### Persistence (Sample 1)

The persistence measure in Sample 1 consisted of a mental rotation task (the “finding game”), in which children were asked to select the rotated version of a target object among three choices (adapted from [36]). The researcher first explained the objective of the task and corrected the child if they selected the wrong object. The researcher then did one warm-up trial and again corrected the child if they selected the wrong object. The researcher then administered four test trials, in which the child was instructed to select from among three options the object that matched the target as quickly as possible. To ensure that performance was equated across children, all four trials contained target objects with no corresponding rotated match. Additionally, to ensure that children questioned their success on the task, the researcher gave explicit negative feedback on the second and fourth trials (i.e., “Try again!”). To assess persistence behavior, at the end of the task, the researcher asked the child whether they wanted to keep playing the finding game or do something else. If the child stated they wanted to keep playing, the researcher asked whether they wanted to do “a few more” trials or “a whole bunch more.” Responses were scored from 0 to 1, with higher numbers reflecting more persistence (0 = *do something else*, 0.5 = *do a few more*, 1 = *do a whole bunch more*).

#### Affective responses to and persistence in the face of failure (Sample 2)

To measure affective responses to and persistence in the face of failure in Sample 2, we administered two role-playing scenarios, during which the participating child was said to have made a mistake while attempting to complete a task (adapted from [37]). To make the task more engaging for the child, the researcher read aloud the scenario while motioning with two plastic figurines, one meant to represent the adult and the other the participating child.

The first scenario involved a story in which the child builds a house out of blocks to give to their teacher but forgets to build windows in the house. In this scenario, the mistake was apparent but did not result in teacher criticism. After reading aloud the first scenario, the researcher asked the child three questions to assess the child’s attitudes toward themselves and the hypothetical product they created (e.g., whether they like it) (see S6 Fig in S2 File in the SOM for items). The second scenario involved a story in which the child paints a picture of a family to give to their teacher but forgets to paint feet on the child. Because prior work has suggested that to experience vulnerability, young children require a very explicit failure experience, such as criticism from an adult (e.g., [32, 34]), in this scenario the mistake resulted in mild criticism from the pretend teacher. The researcher asked the same three questions from the house-building scenario, but with respect to the hypothetical painting. Responses to all six questions were averaged to form an index of the child’s affective response to failure (α = .71 [.65, .76]).

After administering the three affect items for the second scenario, the researcher then administered two questions that assessed children’s persistence (e.g., “If you had a chance to do something tomorrow, would you paint, or would you do something else?”). Responses to the two persistence items were significantly correlated (*r* = .26 [.07, .43], *p* = .007) and were thus combined to form a composite. The two scenarios and the questions after each scenario were always presented in the order described above.

#### Open-ended justifications (Sample 2)

At the end of the sessions, we asked children in Sample 2 to justify a subset of their answers to the GM-C items. The purpose of this measure was to assess whether children who were more versus less growth-oriented, as measured by the GM-C scale, expressed different concepts when asked to justify their responses. If the GM-C scale is a valid measure of mindsets, we would expect children with stronger growth mindsets on it to more often reference concepts that are central to such mindsets—namely, concepts related to change in abilities (forgetting, learning) or strategies for changing abilities (e.g., practicing, effort). To minimize session length, we asked children to justify their responses to only two items: the malleability questions for the low- and high-ability vignettes about math (see S2 Appendix in S2 File for details on the coding scheme).

*Debriefing*. At the end of each session, children received a short debriefing that emphasized the importance of effort for learning and was designed to ensure that children left the session with positive feelings about their participation.

#### Analysis plan

We performed analyses and created figures using R version 4.0.3. We assessed the dimensionality of the scale with EFAs and CFAs using the *psych* (Version 2.1.9; [61]) and *lavaan* (Version 0.6–9; [62]) packages, respectively. We conducted reliability analyses using the *psych* (Version 2.1.9; [61]) and *cocron* (Version 1.0–1; [63]) packages. We assessed the validity of the scale with linear regression models in R’s native package. We created figures using the *ggplot2* package (Version 3.3.5; [64]). We report 95% confidence intervals (*CI*s) in square brackets alongside regression coefficients, odds ratios, alphas, and correlation coefficients. For all tests, *p* values less than the conventional threshold of .05 were considered statistically significant.

#### Open data and analytic syntax

The raw data and analytic syntax for both studies are available on OSF: https://osf.io/hm2yg/?view_only=7da7b40cd6724fae84f1ed3b493f79b7.

### Results and discussion

#### Memory check

The percentage of correct responses to the memory check about whether the protagonist had high versus low ability was near ceiling, with an average of 97% and a median of 100%. Older children performed better on this memory check than younger children, *r*(218) = .14 [.01, .27], *p* = .032.

#### Scale dimensionality

**Fig 1:**
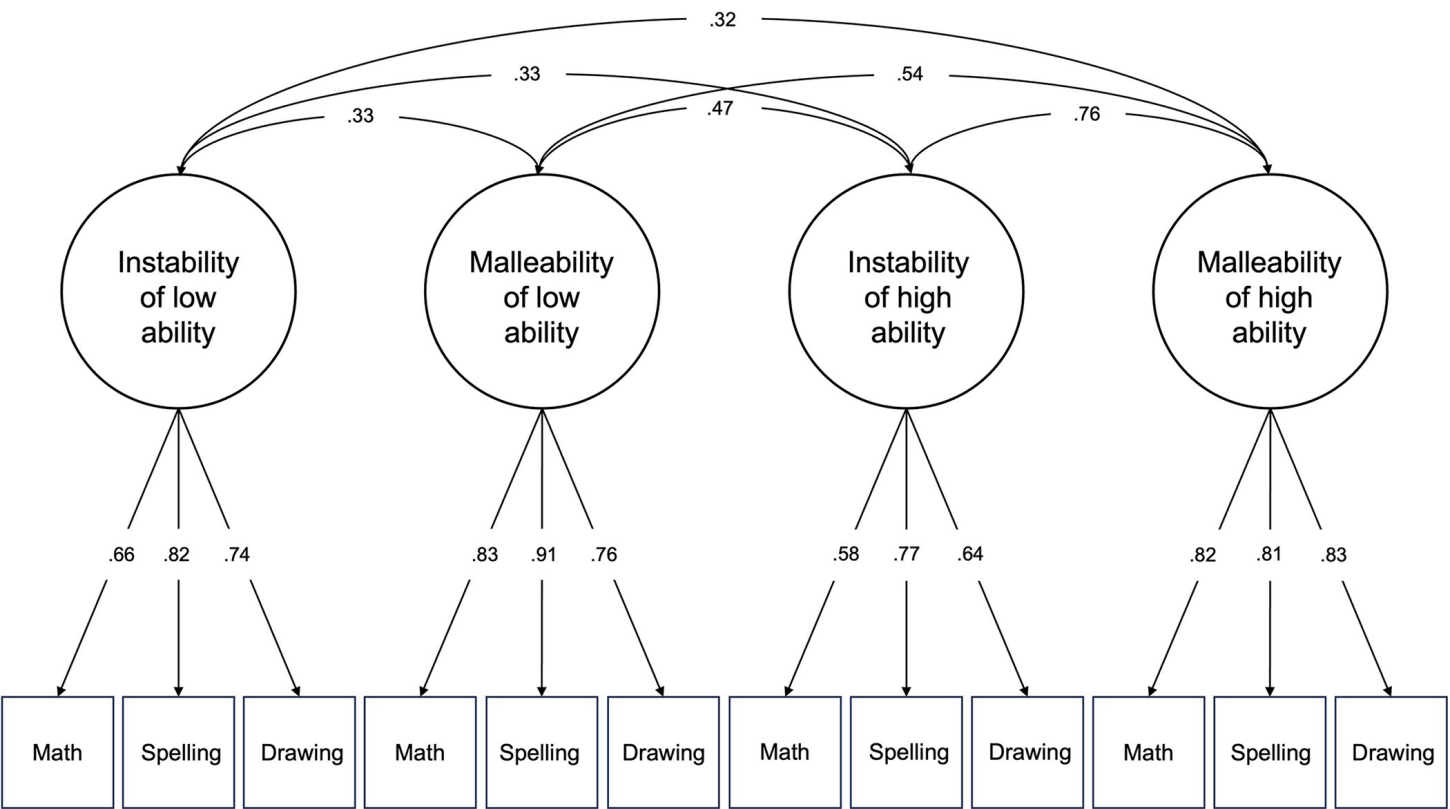
Results of the four-factor CFA. Factor loading and covariance estimates are from the completely standardized solution.

A series of EFAs and CFAs indicated that a four-factor solution (three high-ability instability items, three high-ability malleability items, three low-ability instability items, three low-ability malleability items) provided the best fit to the data: χ^2^(48) = 68.43, *p* = .028; RMSEA = .06; TLI = .95, CFI = .96; SRMR = .05 (see Fig 1 and S1 Appendix and S6 Table in S2 File in the SOM for details). We computed average scale scores for each of the four factors (see S7 Table in S2 File for zero-order correlations between the four scales for Samples 1 and 2 combined). We proceed with reliability and validity analyses using these scale scores.

#### Reliability

Next, using Samples 1 and 2 combined, we evaluated the reliability of each of the four subscales (instability of high ability, malleability of high ability, instability of low ability, and malleability of low ability). First, we assessed internal consistency by calculating Cronbach’s alpha for each subscale, both for the overall sample and separately by age group (ages 4, 5, and 6). Second, we calculated the test-retest reliability of each subscale by correlating each subscale score over time across two time points.

*Internal consistency*. The items comprising each GM-C subscale had internal consistencies that ranged from moderate (α = 0.71) to excellent (α = 0.90) and did not differ across age groups (*p*s > .12). The instability-of-high-ability subscale had the lowest reliability across the scales, especially among 4- and 5-year-olds (see S10 Table in S2 File in the SOM).

*Test-retest reliability*. To assess the reliability of the scale over time, 119 children from the full sample completed the mindset scale again after a delay of approximately one month (*M* = 31 days of lag time, range = 18 to 105 days). Children’s scores on the subscales at Time 1 did not differ by whether they completed the second session (*p*s > .44). We assessed test-retest reliability by computing zero-order Pearson correlations between children’s score on a subscale at Time 1 and their score on that subscale at Time 2. Overall, children’s scores on each of the subscales were reliable over time, but the extent of the test-retest reliability varied across subscales (see S2 Fig in S2 File in the SOM):

- instability of low ability: *r*(117) = .38 [.21, .52], *p* < .001;
- malleability of low ability: *r*(117) = .72 [.62, .79], *p* < .001;
- instability of high ability: *r*(116) = .58 [.45, .69], *p* < .001;
- malleability of high ability: *r*(116) = .54 [.40, .66], *p* < .001.

Although relatively modest by the standards of adult measures, these values are comparable to the test-retest reliabilities of other individual-difference measures assessing children’s social cognition (e.g., [65, 66]).

#### Descriptive statistics

The means of the four GM-C subscales ranged from .29 to .67 (see S7 Table in S2 File in the SOM). The subscale scores were positively correlated, with correlations ranging from .22 to .58 (see S7 Table in S2 File in the SOM). The distributions of the four subscales are displayed in S1 Fig in S2 File in the SOM.

#### Concurrent validity

We next assessed the concurrent validity of the four subscales by evaluating how children’s scores on them relate to their (a) preference for learning (vs. performance) goals, (b) preference for upward (vs. downward) comparisons, (c) persistence, and (d) affective response to failure. In addition, an important goal of these analyses was to arrive at the most concise and robust scale possible. The results of the following analyses indicated that two subscales did not significantly predict scores on any of the measures presented below: specifically, the subscales that measured the instability and malleability of high ability. Conceptually speaking, these items are also a poorer fit with the classic conceptualization of mindsets, which focuses on whether ability *can be increased*. Put differently, the items that assessed children’s beliefs about how much someone’s ability *could grow* were more face-valid than the items that assessed children’s beliefs about how much someone’s ability could atrophy. Therefore, we focus here on how the subscales concerning the instability and malleability of *low* ability relate to theoretically relevant outcomes and present the results of analyses concerning high ability in the SOM (see S16-S20 Tables in S2 File). We examine instability and malleability of low ability as separate constructs, as suggested by the CFAs described above, but also as a unitary construct due to the moderate correlation (*r* = 0.33) between the two latent factors in the final CFA model (see also S7 Table in S2 File in the SOM for the correlation between average subscale scores).

In what follows, the results for validation measures that were common across the two samples are presented together; results for measures that were administered for only one of the samples are presented separately.

*Learning versus performance goals (Samples 1 and 2)*. We first examined the extent to which children’s responses on the GM-C scale predicted their goal orientations on the task in which they decided whether to complete a challenging puzzle (that they would learn from) or an easy puzzle (that they would do well on). We specified logistic regression models with children’s puzzle selection as the outcome, and children’s GM-C subscale scores and age as predictors.

First, children’s six-item GM-C score significantly predicted their choice on this task, such that children with stronger growth mindsets were more likely to select the challenging puzzle (learning goal) (odds ratio = 6.42 [2.15, 20.78], *b* = 1.86 [0.77, 3.03], *SE* = 0.58, *p* = .001). Age was also related to children’s choice on this task, with older children selecting the challenging puzzle (learning goal) more often than younger children (odds ratio = 1.83 [1.26, 2.67], *b* = 0.60 [0.23, 0.98], *SE* = 0.19, *p* = .002).

Next, we examined the relation between children’s subscale scores and their goals. Children’s beliefs about the instability of low ability were a significant predictor of children’s puzzle choice, such that children who thought low ability was more unstable were more likely to choose the challenging puzzle (learning goal): odds ratio = 2.85 [1.26, 6.76], *b* = 1.05 [0.23, 1.91], *SE* = 0.43, *p* = .014 (see S3 Fig in S2 File in the SOM). Children’s scores on the malleability-of-low-ability subscale were also a significant predictor of children’s goals, such that children who viewed low ability as more malleable were more likely to choose the challenging puzzle (learning goal): odds ratio = 3.84 [1.56, 10.12], *b* = 1.35 [0.44, 2.31], *SE* = 0.47, *p* = .005.

When both subscale scores were entered into the model simultaneously along with children’s age, the malleability score remained a significant predictor (odds ratio = 3.06 [1.19, 8.33], *b* = 1.12 [0.17, 2.12], *SE* = 0.49, *p* = .024). The coefficient for the instability subscale became marginal (odds ratio = 2.15 [0.91, 5.28], *b* = 0.77 [−0.10, 1.66], *SE* = 0.45, *p* = .087).

*Upward versus downward comparison*, *persistence*, *and affective response to failure*. Children’s scores on the subscales concerning low ability (considered separately or as a composite) did not correlate with any of the other motivation-related measures (see S12-S15 Tables in S2 File in the SOM).

*Open-ended justifications (Sample 2)*. Children’s scores on the instability-of-low-ability subscale predicted the content of their justifications for their answers to both the high-ability math vignette, *r*(69) = .45 [.25, .62], *p* < .001, and the low-ability math vignette, *r*(74) = .39 [.18, .56], *p* < .001. This means that children who believed that low ability was unstable were also more likely to mention concepts related to processes such as learning, effort, practice, and forgetting (see S11 Table in S2 File in the SOM for sample justifications); in contrast, children who believed that low ability was stable tended to mention concepts related to ability or constancy in cognitive skills such as being (not) good at math, being smart, or remembering. Similarly, children’s scores on the malleability-of-low-ability subscale predicted the content of their justifications for their answers to both the high-ability math vignette, *r*(69) = .54 [.34, .68], *p* < .001, and the low-ability math vignette, *r*(74) = .58 [.41, .71], *p* < .001. Together, these results suggest that (a) in general, children understood how to use the response scales, and (b) children articulated concepts that were in line with their mindsets, as measured by the GM-C.

#### Age differences in mindsets (Samples 1 and 2)

The GM-C scale exhibited strong invariance across age groups (4-, 5-, and 6-year-olds) (see S3 Appendix and S8 Table in S2 File in the SOM for results of the multi-group CFAs). Therefore, we were able to examine whether children’s mindsets change with age. To examine the relation between children’s scores on the two low-ability subscales and their age, we specified two linear regression models. In each, children’s scores on a particular subscale served as the dependent variable, and age (continuous, in years with 2+ decimal precision) served as the predictor. Age was not related to children’s scores on the instability subscale (*p* = .84). For the malleability subscale, older children had stronger growth mindsets (*b* = 0.15 [0.09, 0.20], *SE* = 0.03, *p* < .001; equivalent to an increase of 0.40 standard deviations per year of age).

#### Conclusion

Several important findings emerged from this study. First, the full GM-C scale’s dimensionality appeared to be best represented by a four-factor structure, with factors for the instability of low ability, instability of high ability, malleability of low ability, and malleability of high ability. Second, the items in the four GM-C subscales evidenced acceptable internal consistency, both across the entire sample and in each of the three age groups separately (i.e., 4-, 5-, and 6-year-olds). Third, children’s scores on the four GM-C subscales were moderately reliable across time. Fourth, we found evidence of strong measurement invariance across age groups.

Fifth, the evidence pertaining to the scale’s validity was more mixed. Children’s scores on the subscales that concerned high ability did not predict children’s attitudes and behavior for any of the outcomes included here. It may be that these facets—which concerned the extent to which someone’s high ability may atrophy over time, especially without practice—are not as reflective of the mindset construct as those concerning whether someone’s low ability can grow. That is, it can be argued that beliefs concerning the possibility for one’s ability to improve are more reflective of mindset beliefs as previously theorized [1, 2].

Promisingly, children’s beliefs about the instability and malleability of low ability did predict children’s goal orientations: Children who were more growth-oriented about low ability also gravitated toward learning goals. When scores on both low-ability subscales (instability and malleability) were entered together in a single model, children’s scores on the malleability subscale remained a significant predictor, but their scores on the instability subscale became marginal. Further evidence of the GM-C scale’s validity was provided by children’s explicit justifications for their answers to the scale items, which seemed to genuinely reflect their beliefs about the nature of ability. When asked to justify their responses, children who viewed low ability as more unstable and malleable (i.e., who had high scores on the GM-C) were more likely to reference concepts related to processes (e.g., learning, effort, practice, forgetting). In contrast, children who viewed low ability as less unstable and malleable (i.e., who had low scores on the GM-C) referenced concepts related to constancy (e.g., being not good, being smart, remembering).

However, not all results supported the conclusion that the low-ability GM-C subscales were valid: Children’s scores on these subscales were unrelated to their persistence behavior, tendency to engage in upward comparisons, and their affective responses to failure. It is not altogether clear how to interpret these results. On the one hand, perhaps this evidence speaks against the validity of the GM-C scale. On the other hand, given that mindsets are underexplored in this young age group, this evidence may instead reveal that young children’s motivational frameworks have not yet coalesced (e.g., [56]). Study 2 allowed us to (a) get some purchase on this question by including a sample of slightly older children and (b) assess the properties of the GM-C scale in a different cultural context (namely, in South Africa).

## Study 2

The goals of Study 2 were three-fold. First, we assessed the validity of the GM-C measure in a sample of slightly older elementary-school-aged children (in Grades 2–5). Recruiting older children allowed us to investigate whether the GM-C scale relates to other elements of children’s motivational frameworks in an age range where we can be more confident that these relations should be present. Relatedly, recruiting older children also allowed us to examine the links between scores on the GM-C and children’s *academic achievement* (namely, their grades in school). Prior work with older samples of children has suggested that students with growth mindsets earn better grades if they are facing academic risks (e.g., [3, 6, 18, 48]). The children in our sample faced multiple academic risks as residents of areas with substantial resource constraints, including lack of funding for school infrastructure and materials [67]. Second, we assessed the cross-cultural robustness of the GM-C scale by recruiting a large sample of children living in the Western Cape, South Africa. Third, we assessed the convergent validity of the GM-C scale by also measuring children’s mindsets using an existing scale suitable for older children [1].

### Method

#### Participants

A total of 331 children answered the items in the GM-C scale. Participants were Xhosa- and Afrikaans-speaking children attending no-fee after-school programs serving low-resourced schools in the Western Cape, South Africa. Children participated between April 2018 and May 2018. Most of the children were fourth (*n* = 103) or fifth (*n* = 104) graders, and ten were second (*n* = 4) or third (*n* = 6) graders; grade information was missing for 34% of this sample. Of the students for whom gender information was available, 54% of children were girls and 46% were boys; gender data were missing for 79% of the sample. Children represented eight schools; school information was missing for 34% of the sample. We did not collect data on children’s socio-economic status or race and ethnicity directly (typical US American questionnaire items assessing these characteristics were not appropriate for this context); our description of the sample as consisting of children who were non-White and had low socio-economic status is based on the fact that all children lived in severely under-resourced townships (the communities for which the after-school programs were developed) [67].

#### Materials and procedure

Due to the conceptual and validity issues that surfaced in Study 1 with respect to the high-ability GM-C subscales, in Study 2 we only administered the subscales concerning low ability, which also helped to minimize the length of the study—a concern for school officials.

*The GM-C measure*. Children received a shortened version of the GM-C scale from Study 1, consisting only of the instability- and malleability-of-low-ability subscales (3 domains × 2 questions = 6 items total). Facilitators with basic proficiency in Afrikaans, English, and Xhosa administered paper surveys to children in small groups (each child completed their own survey) (see S4 Appendix in S2 File for additional methodological details). Similar to Study 1, we formed two subscales by averaging children’s responses to the three instability-of-low-ability items and, separately, the three malleability-of-low-ability items.

*Validation measures*. As in Study 1, we assessed children’s learning goals. Further, we assessed children’s challenge-seeking behavior, as well as their achievement in mathematics and English. We were not able to include some of the other validation measures from Study 1 because of concerns about the length of the study. We expected that children who were more growth-oriented on the GM-C scale would be more likely to orient toward learning (rather than performance) goals, seek out challenges, and earn better grades in math and English.

Because this sample of children was slightly older, we also administered an adapted version of the classic mindset (or theory of intelligence) scale [1, 2] to evaluate how children’s scores on it relate to their scores on the GM-C scale. Finally, to assess incremental validity, we examined whether children’s scores on the GM-C scale predicted the other components of their motivational frameworks beyond their scores on the classic scale.

#### Learning goals

The task assessing learning versus performance goals was identical to that in Study 1. One child’s response was missing.

#### Challenge-seeking

In the challenge-seeking task, children first completed one Raven’s progressive matrix (which is essentially a pattern-matching puzzle) and were then asked, “For this next puzzle, would you like to do one that is on the easy side, or on the challenging side?” (*very easy* = 0, *easy* = 0.25, *medium* = 0.50, *challenging* = 0.75, *very challenging* = 1.00). Two children’s responses were missing.

#### Math and English achievement

Children’s achievement in math and English was measured via report card grades (on a 1–7 scale) collected from official school records at the end of the school year. The average math grade was 4.76, roughly a C; the average English grade was 4.03, roughly a C. Math grades were missing for 35% of the sample; English grades were missing for 34% of the sample.

#### The theory of intelligence measure

Children responded to four items assessing their entity versus incremental theory of intelligence (TOI). Three items were adapted from Dweck’s original TOI scale (e.g., “You can learn new things, but you cannot make yourself smarter”; [1, 2]), and one item was developed more recently and used in a previous study (i.e., “People are born smart or not smart. This can’t be changed”; [68]). All four items concerned fixed mindsets, and children indicated their endorsement on a five-point scale (*completely true* = 0, *a little true* = 0.25, *both* = 0.50, *a little not true* = 0.75, *not at all true* = 1). The four items demonstrated acceptable internal consistency (α = .62 [.56, .69]), so they were averaged to form a composite score, with higher numbers reflecting a stronger growth mindset (*M* = .41, *SD* = 0.27).

### Results and discussion

#### Memory check

The percentage of correct responses on the memory check was again high, with an average of 89% and a median of 100%. There was no relation between children’s grade level and their accuracy on the memory checks, *r*(215) = −.01 [−.14, .13], *p* = .94.

#### Internal consistency

The items comprising each subscale had internal consistencies that ranged from moderate to excellent: αs = .70 [.65, .76] and .90 [.88, .92] for the instability and malleability subscales, respectively. The internal consistency of the items within a subscale for the two grades considered separately (fourth and fifth) was acceptable for the instability subscale and excellent for the malleability subscale (see S21 Table in S2 File in the SOM).

#### Preliminary analyses

The means on the instability and malleability subscales were .81 (*SD* = 0.26) and .75 (*SD* = 0.30), respectively. The two subscales were not correlated, *r*(329) = −.01 [−.12, .09], *p* = .80. The distributions of the two subscales are depicted in S8 Fig in S2 File in the SOM.

#### Concurrent and convergent validity

We next tested the concurrent and convergent validity of the two subscales by evaluating how they relate to children’s (a) preference for learning goals, (b) challenge-seeking behavior, (c) math grades, (d) English grades, and (e) scores on the “classic” measure of mindsets (or TOI), suitable for older children. Because the two GM-C subscales were uncorrelated in this sample, we examined them as separate constructs.

*Learning versus performance goals*. We specified a logistic regression model with children’s choice of puzzle as the dependent variable. When both GM-C subscale scores were entered into the model simultaneously along with grade level, both the instability score (odds ratio = 1.40 [1.08, 1.80], *b* = 0.33 [0.08, 0.59], *SE* = 0.13, *p* = .011) and the malleability score (odds ratio = 1.42 [1.15, 1.75], *b* = 0.35 [0.14, 0.56], *SE* = 0.11, *p* = .001) were significant predictors of children’s goals, such that children who viewed low ability as more unstable and malleable were more likely to select the challenging puzzle (learning goal; see S10 Fig in S2 File). Students’ grade level did not predict their preference for a challenging puzzle (*p* = .33). Finally, even after adjusting for children’s TOI score, children’s scores on the instability (*p* = .019) and the malleability (*p* = .001) subscales remained significant predictors of children’s goals. TOI scores did not relate to goals (*p* = .21).

*Challenge-seeking*. We specified a linear regression model with children’s puzzle choice as the dependent variable. When both subscale scores were entered into the model simultaneously along with grade level, children’s score on the instability subscale was a significant predictor of their puzzle choice (*b* = 0.26 [0.08, 0.45], *β* = 0.19, *SE* = 0.09, *p* = .006), such that children who viewed low ability as less stable were more likely to select a challenging puzzle. Neither children’s score on the malleability subscale (*p* = .48) nor their grade level (*p* = .30) were significant predictors of their choice (see S11 Fig in S2 File). After including children’s TOI score in the model as a predictor, children’s score on the instability subscale remained a significant predictor (*p* = .015). TOI scores also predicted children’s choice, such that children who were more growth-oriented, as measured by the TOI scale, were more likely to select a challenging puzzle (*b* = 0.21 [0.03, 0.39], *β* = 0.16, *SE* = 0.09, *p* = .020).

*Math achievement*. We specified a linear regression model with children’s math grade as the dependent variable. When both GM-C subscale scores were entered into the model simultaneously along with grade level, children’s score on the instability subscale was a significant predictor of their math achievement (*b* = 2.06 [1.17, 2.95], *β* = 0.30, *SE* = 0.45, *p* < .001), such that children who viewed low ability as less stable earned higher grades in math (see S12 Fig in S2 File in the SOM). Children’s score on the malleability subscale was not a significant predictor of their math achievement (*p* = .96). Additionally, older children earned lower grades in math than younger children did (*b* = −0.69 [−1.04, −0.33], *β* = −0.25, *SE* = 0.18, *p* < .001). Finally, after including children’s TOI score in the model as a predictor, children’s score on the instability subscale remained a significant predictor (*p* < .001). Children’s TOI scores also predicted their math achievement, such that children who were more growth-oriented earned higher grades in math (*b* = 1.55 [0.71, 2.38], *β* = 0.23, *SE* = 0.42, *p* < .001).

*English achievement*. We specified a linear regression model with children’s English grade as the dependent variable. When both GM-C subscale scores were entered into the model simultaneously along with grade level, children’s score on the instability subscale was a significant predictor of their English achievement (*b* = 1.64 [0.75, 2.53], *β* = 0.21, *SE* = 0.45, *p* < .001), such that children who viewed low ability as less stable earned higher grades in English (see S13 Fig in S2 File in the SOM). Children’s score on the malleability subscale was not a significant predictor of their English achievement (*p* = .85). Additionally, older children earned lower grades in English than younger children did (*b* = −1.67 [−2.02, −1.31], *β* = −0.54, *SE* = 0.18, *p* < .001). Finally, after including children’s TOI score in the model as a predictor, the instability subscale remained a significant predictor (*p* < .001). TOI scores did not predict English achievement (*p* = .87).

*Theory of intelligence*. We next examined how children’s scores on the two subscales of the GM-C correlated with their scores on the “classic” Likert-type mindset scale [1,2]. The instability subscale correlated with the TOI scale, albeit modestly, *r*(329) = .16 [.05, .26], *p* = .004, such that children who expressed stronger growth mindsets on our mindset scale also reported stronger growth mindsets on the TOI scale. The malleability subscale did not correlate with the TOI scale, *r*(329) = .06 [−.05, .17], *p* = .29.

#### Age differences in mindsets

To examine the relation between children’s scores on the two subscales and their grade we specified two linear regression models. In each, one of the subscales served as the dependent variable, and grade level served as the predictor. Grade level significantly predicted children’s scores on the instability subscale, with older children endorsing stronger growth mindsets than younger children (*b* = 0.06 [0.01, 0.12], *SE* = .03, *p* = .016; equivalent to an increase of 0.25 standard deviations per grade). Grade level was not related to children’s scores on the malleability subscale (*p* = .73).

#### Conclusion

The results of Study 2 provide additional evidence for the reliability and validity of the GM-C scale. As in Study 1, the internal consistency of the two GM-C subscales (instability and malleability of low ability) was acceptable. In addition, children’s scores on one or both of the GM-C subscales predicted children’s preference for learning (vs. performance) goals, their challenge-seeking behavior, as well as their math and English grades, even when adjusting for children’s scores on Dweck’s original TOI scale [1]. These results demonstrate the validity of the GM-C measure. When compared with the results on the younger sample in Study 1, they also suggest that children’s mindsets do in fact become more tightly linked with other elements of their motivational frameworks over development, though this comparison is complicated by the differences in the cultural context of the two samples and by the differences in the motivational and achievement outcomes included across studies. Notably, children’s scores on the GM-C subscales were more consistently predictive of children’s motivational and achievement outcomes than their scores on the standard TOI scale, which did not predict children’s learning (vs. performance) goals or their grades in English in this study. Finally, these results offer evidence for the cross-cultural robustness of the GM-C scale.

## General discussion

Mindsets are an important component of people’s motivational frameworks. A growing body of evidence points to the relevance of these beliefs even for very young children. The goal of the present research was to develop a mindset scale suitable for young children and assess its psychometric properties. Specifically, the present studies aimed to assess (a) the dimensionality, (b) measurement invariance, (c) internal consistency, (d) temporal stability, (e) concurrent validity, and (f) cross-cultural robustness of the new GM-C scale. We found that the GM-C exhibited four factors: beliefs about the instability of low ability, beliefs about the malleability of low ability, beliefs about the instability of high ability, and beliefs about the malleability of high ability. The low-ability items of the GM-C—which we recommend based on the considerations summarized next—had adequate psychometric properties, including invariance across age, acceptable internal consistency and moderate temporal stability over approximately one month. We also found evidence in support of these items’ concurrent validity: Children’s scores on the low-ability subscales predicted their goal orientations (Studies 1 and 2), challenge-seeking behavior, as well as their math and English achievement (Study 2).

Based on the results of these studies, we recommend that researchers use the six low-ability items of the GM-C only—both because they are more face-valid than the high-ability items and because they had more desirable psychometric properties. A further question about the use of the GM-C scale concerns whether to collapse across the two low-ability subscales (instability and malleability) or keep them separate. Because these subscales correlated in Study 1 but not in Study 2, we cannot make firm recommendations on this point; researchers should base this decision on what they observe in their sample. A six-item composite score may be used if the instability- and malleability-of-low-ability subscales correlate significantly. If they do not, we recommend treating the two subscales as separate constructs. In what follows, except where noted, we discuss the results concerning the low-ability subscales only.

Several psychometric properties of the GM-C scale are promising. In Study 1, the internal consistency of the instability- and malleability-of-low-ability subscales was strong overall and within each of the three age groups (4-, 5-, and 6-year-olds). In Study 2, the malleability-of-low-ability subscale items demonstrated strong internal consistency, both overall and among fourth and fifth graders considered separately. The instability-of-low-ability subscale items demonstrated acceptable internal consistency overall and among fourth and fifth graders separately. Together, these results suggest that the items comprising these subscales (and the constructs they are tapping) can be considered homogenous.

In Study 1, the test-retest reliabilities of the instability- and malleability-of-low-ability subscales were .38 and .72, respectively. These values are similar (or larger) in magnitude to the test-retest reliabilities of other measures of individual differences in children’s social cognition (e.g., [65, 66]). Thus, it seems reasonable to conclude that these subscales are tapping a stable construct. However, future work may assess the temporal stability of the measure across different intervals (e.g., longer than one month) to gain a clearer understanding of the reliability of the GM-C scale over time.

Support for the GM-C scale’s validity was provided by the fact that scores on the low-ability subscales predicted children’s goal orientations (Studies 1 and 2), challenge-seeking behavior (Study 2), and their math and English achievement (Study 2). Also, in Study 1, children articulated concepts in their open-ended justifications that were indicative of the sort of mindset they espoused on the GM-C. That is, children with growth mindsets, as measured by the GM-C, were more likely to cite concepts associated with change in abilities (forgetting, learning) or strategies for changing abilities (practicing, effort). In addition, the results suggested that the GM-C scale performs well across contexts. However, because the children in the South Africa sample were older than the children in the US sample, future work may fruitfully examine the psychometric properties of the GM-C scale among younger children outside the US.

Taken together, these findings are important from a practical standpoint. Students’ willingness to embrace and master challenging academic material is critical to their learning and success in school. Because these behaviors are tied to students’ ability-related beliefs, the present findings may have practical implications for identifying maladaptive beliefs about ability and developing interventions to combat negative effects of such beliefs. The GM-C is a useful, brief instrument for measuring individual differences in mindsets among children as young as four.

In addition to documenting the psychometric features of the GM-C scale, these studies revealed several findings with implications for theory. The first concerns age-related change in children’s mindsets: In both studies, older children tended to espouse stronger growth mindsets compared to younger children. This was so even though the two studies focused on different age groups: 4- to 6-year-olds in Study 1 and 7- to 11-year-olds in Study 2. Notably, however, and consistent with prior work (e.g., [44, 45]), the magnitude of this age-related change depended on the precise belief and age range being tested. Among 4- to 6-year-olds (Study 1), older children endorsed the idea that ability was malleable (i.e., more responsive to intervention) more strongly than younger children did, but there was no age-related change in children’s ideas about the *stability* of ability across time. Among second through fifth graders (Study 2), however, older children endorsed the idea that ability was both malleable (albeit marginally) and unstable across time more strongly than younger children did.

The general picture that older children espouse stronger growth mindsets compared to younger children may seem somewhat surprising: It is generally thought that younger children have strongly optimistic self-views (e.g., [69]) and beliefs about the possibility for change in human attributes (e.g., [58]). Further, as children move through preschool and elementary school, ability-related cues (e.g., formal assessment, ability grouping) become increasingly salient in their environments—and such changes have led some to theorize that children’s mindsets may become more fixed with age (e.g., [32]). However, as reviewed in the introduction, prior evidence on age-related changes in children’s beliefs about the malleability of intelligence is actually mixed: Some studies have found no age-related changes across the early elementary school years [46, 47], whereas others have found—similar to the results reported here—that children’s beliefs tend to become more growth-oriented in the later elementary school years [13, 44]. We tentatively conclude that the weight of the evidence now favors this second possibility: that children’s mindsets become more growth-oriented with age. This conclusion is indirectly supported by a rich body of research on children’s causal-explanatory reasoning, which has documented that, with age, children become increasingly aware of the extent to which others’ behaviors and psychological traits are shaped by their environments rather than being immutable (e.g., [70–72]). We look forward to more research testing our claim that children’s growth mindsets become stronger over development.

The present results also provide insights into the coherence of children’s motivational frameworks across development. Consistent with some prior evidence (e.g., [47]), we found that children’s mindsets, as measured with the GM-C scale, showed stronger relationships with the rest of their motivational frameworks in older children (Study 2) compared to younger children (Study 1). On the one hand, this result is unsurprising considering that these components hang together well among adults (e.g., [8]). Thus, it is sensible to expect that the degree of coherence would increase across childhood. On the other hand, the prior literature was not entirely consistent on this point (e.g., [27]), so the present findings contribute a reassuring bit of evidence. We would note, however, that the age comparison here is complicated by the other differences between the samples in Studies 1 and 2, as we discuss next.

Another noteworthy and theoretically relevant finding concerns cross-cultural differences in children’s growth mindsets. Children in Study 2 espoused stronger growth mindsets (on both subscales of the measure) than children in Study 1 did. One reason may be that children in Study 2 were older than children in Study 1: As just discussed, the results of the present studies and some prior work (e.g., [13, 44]) point to stronger growth mindsets in older children. Another, not mutually exclusive, possibility is that this difference reflects a true cross-cultural difference in growth mindset endorsement. Although cross-cultural work on mindsets is somewhat limited, one recent study found that compared to Chinese students, US American students tended to espouse stronger growth mindsets [54]. Other work has similarly found stronger growth mindsets among (a) teachers raised in the UK (versus teachers raised in East Asia) [73] and (b) US American parents (compared to parents in New Zealand, China, and Japan) [74]. Because the present data cannot shed light on the precise source of these differences, future studies should disentangle the contribution of age versus culture to young children’s mindsets.

### Outstanding questions, future directions, and limitations

Several important questions that may be considered for future investigations remain. One concerns the slightly different pattern of results for the instability- and malleability-of-low-ability subscales across studies. In Study 1, the malleability subscale demonstrated better psychometric properties than the instability subscale: It had stronger internal consistency, better test-retest reliability, and was more predictive of children’s goal orientations. However, in Study 2, the instability subscale more consistently predicted children’s achievement behavior and grades, but it also had slightly lower internal reliability compared to the malleability subscale. This pattern of findings suggests that sometimes beliefs about the *instability* of ability are more closely involved in motivation-relevant reasoning and behavior, while other times it is beliefs about the *malleability* of ability that drive motivation-relevant reasoning and behavior. It is also unclear why the two subscale scores were correlated in Study 1 but not in Study 2 (but see [13] for a similar finding). More research is needed to better understand how these constructs relate to each other and to behavior, vary with age, and differ between cultural contexts.

Relatedly, the results of the present studies indicated that beliefs about the instability and malleability of *high* ability are empirically distinct from beliefs about the instability and malleability of *low* ability. Although beliefs about the extent to which high ability can atrophy may not be at the core of mindsets as currently conceptualized, these beliefs are interesting in and of themselves, and we await future work that may clarify their meaning and correlates.

An important limitation of the present set of studies is that our approach for evaluating the validity of the scale involved concurrent associations only. Thus, further investigation will be needed to assess the GM-C scale’s utility in predicting children’s *future* outcomes. Another limitation concerns our approach to developing the scale in Study 2. Although the inclusion of non-White South African participants in the development of the GM-C scale is noteworthy, inclusion of participants from non-Western cultures should ideally occur at an even earlier step in the scale development process: Mindset research—and scale development efforts more broadly—would benefit enormously from careful examination of related constructs in non-Western samples. Concretely, this could involve conducting open-ended interviews with participants so that researchers can arrive at a culturally informed conceptualization of the constructs they are studying *before* operationalizing them via item generation. An approach of this sort would go beyond mere inclusion of diverse samples of participants in research that concerns constructs that were conceived for and by Western, majority-group members.

### Conclusion

Mindsets are an important piece of children’s motivational frameworks, with significant consequences for children’s learning and achievement in school. The results of the present studies demonstrate that mindsets are both a meaningful and a measurable construct among young children. The instrument we developed is a useful tool for measuring individual differences in young children’s beliefs about the malleability of intelligence.

## Supporting information

S1 File Copy of the GM-C scale. (PDF)

S2 File Supplemental online material. (DOCX)
