# Supplementary material for: A Growth Mindset Scale for Young Children (GM-C): Development and validation among children from the United States and South Africa
Source: PLoS One. 2024 Oct 7;19(10):e0311205. doi: 10.1371/journal.pone.0311205 (PMC11458031; doi:10.1371/journal.pone.0311205)
Supplement: S1 File — (PDF) [file pone.0311205.s001.pdf]

<u>**Introduction**</u>

In this game, I'm going to tell you some things about some kids. When we talk about these kids, make sure you pay close attention, because I'll ask you some questions about these kids. Okay?

A. [*show picture of character]* This is Alex. And here's something about Alex: Alex is really good at math. *[show green check mark and numbers]* Alex gets *all* of the math problems right on her schoolwork.

I just want to make sure you were paying attention: Is Alex good at math? Or not good at math?

[circle one]: **Good / Not good**

If "good": That's right, Alex is really good at math.
If "not good": Actually, Alex is really good at math.

[remove green check mark and numbers from view]

Now here's a question for you: Will it *always* be this way? Will Alex *always* be really good at math?

[circle one]: **Yes / No**

How sure are you about this? Are you sort of sure? Or really sure?

[circle one]: **Sort of / Really**

Now let me tell you what happened with Alex. When Alex was a little older, she moved to a school far away. At this school, kids don't do a lot of math. After Alex started at this far-away school, she didn't get to practice math very much. Alex didn't do almost any math at this school.

Now here's a question for you: Alex was at this school for a long time. When she left this school, was she good at math or not good at math?

[circle one]: **Good / Not good**

If "good" [use smiley scale]: Was she sort of good, good, or really good?

[circle one]: **Sort of good / good / really good**

If "not good" [use frowny scale]: Was she sort of not good, not good, or really not good?

[circle one]: **Sort of not good / not good / really not good**

B. [*show picture of character]* This is Pat. And here's something about Pat: Pat is really good at spelling. *[show green check mark and letters]* Pat gets *all* of the spelling questions right on his schoolwork.

I just want to make sure you were paying attention: Is Pat good at spelling? Or not good at spelling?

[circle one]: **Good / Not good**

If "good": That's right, Pat is really good at spelling.
If "not good": Actually, Pat is really good at spelling.

[remove green check mark and letters from view]

Now here's a question for you: Will it *always* be this way? Will Pat *always* be really good at spelling?

[circle one]: **Yes / No**

How sure are you about this? Are you sort of sure? Or really sure?

[circle one]: **Sort of / Really**

Now let me tell you what happened with Pat. When Pat was a little older, he moved to a school far away. At this school, kids don't do a lot of spelling. After Pat started at this far-away school, he didn't get to practice spelling very much. Pat didn't spend very much time spelling words at this school.

Now here's a question for you: Pat was at this school for a long time. When he left this school, was he good at spelling or not good at spelling?

[circle one]: **Good / Not good**

If "good" [use smiley scale]: Was he sort of good, good, or really good?

[circle one]: **Sort of good / good / really good**

If "not good" [use frowny scale]: Was he sort of not good, not good, or really not good?

[circle one]: **Sort of not good / not good / really not good**

C. [*show picture of character]* This is Sam. And here's something about Sam: Sam is really good at drawing. *[show green check mark and easel]* Sam can draw *anything* her teacher asks her to.

I just want to make sure you were paying attention: Is Sam good at drawing? Or not good at drawing?

[circle one]: **Good / Not good**

If "good": That's right, Sam is really good at drawing.
If "not good": Actually, Sam is really good at drawing.

[remove green check mark and easel from view]

Now here's a question for you: Will it *always* be this way? Will Sam *always* be really good at drawing?

[circle one]: **Yes / No**

How sure are you about this? Are you sort of sure? Or really sure?

[circle one]: **Sort of / Really**

Now let me tell you what happened with Sam. When Sam was a little older, she moved to a school far away. At this school, kids don't do a lot of drawing. After Sam started at this far-away school, she didn't get to practice drawing very much. Sam didn't spend very much time drawing at this school.

Now here's a question for you: Sam was at this school for a long time. When she left this school, was she good at drawing or not good at drawing?

[circle one]: **Good / Not good**

If "good" [use smiley scale]: Was she sort of good, good, or really good?

[circle one]: **Sort of good / good / really good**

If "not good" [use frowny scale]: Was she sort of not good, not good, or really not good?

[circle one]: **Sort of not good / not good / really not good**

D. [*show picture of character]* This is Jamie. And here's something about Jamie: Jamie isn't very good at math. *[show red X mark and numbers]* Jamie gets *a lot* of math problems wrong on her schoolwork.

I just want to make sure you were paying attention: Is Jamie good at math? Or not good at math?

[circle one]: **Good / Not good**

If "good": Actually, Jamie isn't very good at math.
If "not good": That's right, Jamie isn't very good at math.

[remove red X and numbers from view]

Now here's a question for you: Will it *always* be this way? Will Jamie *always* be not very good at math?

[circle one]: **Yes / No**

How sure are you about this? Are you sort of sure? Or really sure?

[circle one]: **Sort of / Really**

Now let me tell you what happened with Jamie. When Jamie was a little older, she moved to a school far away. At this school, kids do a lot of math. After Jamie started at this far-away school, she got to practice math a lot. Jamie did a lot of math at this school.

Now here's a question for you: Jamie was at this school for a long time. When she left this school, was she good at math or not good at math?

[circle one]: **Good / Not good**

If "good" [use smiley scale]: Was she sort of good, good, or really good?

[circle one]: **Sort of good / good / really good**

If "not good" [use frowny scale]: Was she sort of not good, not good, or really not good?

[circle one]: **Sort of not good / not good / really not good**

E. [*show picture of character]* This is Kendall. And here's something about Kendall: Kendall isn't very good at spelling. *[show red X and letters]* Kendall gets *a lot* of spelling questions wrong on his schoolwork.

I just want to make sure you were paying attention: Is Kendall good at spelling? Or not good at spelling?

[circle one]: **Good / Not good**

If "good": Actually, Kendall isn't very good at spelling.
If "not good": That's right, Kendall isn't very good at spelling.

[remove red X and letters from view]

Now here's a question for you: Will it *always* be this way? Will Kendall *always* be not very good at spelling?

[circle one]: **Yes / No**

How sure are you about this? Are you sort of sure? Or really sure?

[circle one]: **Sort of / Really**

Now let me tell you what happened with Kendall. When Kendall was a little older, he moved to a school far away. At this school, kids do a lot of spelling. After Kendall started at this far-away school, he got to practice spelling a lot. Kendall spent a lot of time spelling words at this school.

Now here's a question for you: Kendall was at this school for a long time. When he left this school, was he good at spelling or not good at spelling?

[circle one]: **Good / Not good**

If "good" [use smiley scale]: Was he sort of good, good, or really good?

[circle one]: **Sort of good / good / really good**

If "not good" [use frowny scale]: Was he sort of not good, not good, or really not good?

[circle one]: **Sort of not good / not good / really not good**

F. [*show picture of character]* This is Casey. And here's something about Casey: Casey isn't very good at drawing. *[show red X and easel]* Casey can not draw *anything* her teacher asks her to.

I just want to make sure you were paying attention: Is Casey good at drawing? Or not good at drawing?

[circle one]: **Good / Not good**

If "good": Actually, Casey isn't very good at drawing.
If "not good": That's right, Casey isn't very good at drawing.

[remove red X and easel from view]

Now here's a question for you: Will it *always* be this way? Will Casey *always* be not very good at drawing?

[circle one]: **Yes / No**

How sure are you about this? Are you sort of sure? Or really sure?

[circle one]: **Sort of / Really**

Now let me tell you what happened with Casey. When Casey was a little older, she moved to a school far away. At this school, kids do a lot of drawing. After Casey started at this far-away school, she got to practice drawing a lot. Casey spent a lot of time drawing at this school.

Now here's a question for you: Casey was at this school for a long time. When she left this school, was she good at drawing or not good at drawing?

[circle one]: **Good / Not good**

If "good" [use smiley scale]: Was she sort of good, good, or really good?

[circle one]: **Sort of good / good / really good**

If "not good" [use frowny scale]: Was she sort of not good, not good, or really not good?

[circle one]: **Sort of not good / not good / really not good**
