# Supplementary material for: A Growth Mindset Scale for Young Children (GM-C): Development and validation among children from the United States and South Africa
Source: PLoS One. 2024 Oct 7;19(10):e0311205. doi: 10.1371/journal.pone.0311205 (PMC11458031; doi:10.1371/journal.pone.0311205)
Supplement: S2 File — (DOCX) [file pone.0311205.s002.docx]

**Supplementary Online Materials**

***A Growth Mindset Scale for Young Children (GM-C): Development and Validation among Children from the United States and South Africa***

**Table of Contents**

**Appendix S1**: Detailed results of and procedure for EFAs and CFAs (Study 1) 3

**Appendix S2**: Coding scheme for children’s open-ended justifications (Study 1) 6

**Appendix S3**: Measurement invariance across age groups and gender (Study 1) 7

**Appendix S4**: Additional methodological details of scale administration (Study 2) 9

**Appendix S5**: Validation analyses using a simplified malleability subscale (Study 2) 11

**Figure S1:** Ridgeline plots of subscale distributions (Study 1) 14

**Figure S2:** Scatterplots of children’s subscale scores at time 1 and time 2 (Study 1) 15

**Figure S3**: Relation between goals and subscale scores on the GM-C (Study 1) 16

**Figure S4**: Distribution of responses (goals, social comparison, persistence #1) in Study 1 17

**Figure S5**: Distribution of responses (affective response, Scenario 1) in Study 1 18

**Figure S6**: Distribution of responses (affective response, Scenario 2) in Study 1 19

**Figure S7**: Distribution of responses (persistence #2) in Study 1 20

**Figure S8**: Ridgeline plots of subscale distributions (Study 2) 21

**Figure S9**: Distribution of responses (grades, goals, challenge seeking) in Study 2 22

**Figure S10**: Relation between goals and subscale scores on the GM-C in Study 2 23

**Figure S11**: Relation between challenge-seeking and subscale scores on the GM-C in Study 2 24

**Figure S12**: Relation between math achievement and subscale scores on the GM-C in Study 2 25

**Figure S13**: Relation between English achievement and subscale scores on the GM-C in Study 2 26

**Table S1:** Example items from GM-C measure 27

**Table S2**: Item loadings and descriptive statistics for one-factor EFA solution 28

**Table S3**: Item loadings and descriptive statistics for two-factor EFA solution 29

**Table S4**: Item loadings and descriptive statistics for three-factor EFA solution 30

**Table S5**: Item loadings and descriptive statistics for four-factor EFA solution 31

**Table S6**: Fit indices from the confirmatory factor analyses (Study 1) 32

**Table S7**: Means, SDs, and correlations among the four subscales of the GM-C scale (Study 1) 33

**Table S8**: Testing for measurement invariance across age groups (Study 1) 34

**Table S9**: Testing for measurement invariance across gender groups (Study 1) 35

**Table S10**: Cronbach’s alphas overall and by age for subscales of the GM-C (Study 1) 36

**Table S11**: Examples of open-ended justifications (Study 1) 37

**Table S12**: Model predicting persistence from instability and malleability of low ability (Study 1) 38

**Table S13**: Model predicting upward comparison from instability and malleability of low ability (Study 1) 39

**Table S14**: Model predicting failure response from instability and malleability of low ability (Study 1) 40

**Table S15**: Model predicting persistence #2 from instability and controllability of low ability (Study 1) 41

**Table S16**: Model predicting goals from instability and malleability of high ability (Study 1) 42

**Table S17**: Model predicting persistence from instability and malleability of high ability (Study 1) 43

**Table S18**: Model predicting upward comparison from instability and malleability of high ability (Study 1) 44

**Table S19**: Model predicting failure response from instability and malleability of high ability (Study 1) 45

**Table S20**: Model predicting persistence #2 from instability and controllability of high ability (Study 1) 46

**Table S21**: Cronbach’s alphas overall and by grade for subscales of the GM-C (Study 2) 47

**Appendix S1**

**Detailed Results of and Procedure for EFAs and CFAs (Study 1)**

**Exploratory Factor Analysis (Sample 1).** A parallel analysis of the 12 items and an initial examination of a scree plot using Sample 1 provided evidence for a three-factor solution. The first, second, and third factors had eigenvalues of 5.24, 1.97, and 1.52, respectively. To be comprehensive, we assessed one-, two-, three-, and four-factor solutions.

We conducted EFAs, specifying one, two, three, or four factors to be extracted using maximum likelihood (ML) estimation and the oblimin (oblique) rotation method to allow the factors to be correlated (see Tables S2-S5). We consulted the Tucker-Lewis Index (TLI; Tucker & Lewis, 1973) and the Root Mean Square Error of Approximation (RMSEA; Steiger & Lind, 1980) to evaluate model fit. Values of TLI greater than or equal to 0.95 and values of RMSEA less than or equal to 0.06 suggest excellent model fit (Hu & Bentler, 1999).

The one-factor solution provided a poor fit to the data: TLI = .44 and RMSEA = .24. However, all items had factor loadings of .31 or higher, with the exception of one item loading that was .29. The two-factor solution also provided a poor fit to the data: TLI = .66 and RMSEA =.19. Factor loadings for this model ranged between .21 and .93. The three-factor solution provided adequate fit to the data: TLI = .94 and RMSEA = .08. All items had factor loadings of .34 or higher, with the exception of one item loading that was .27. A four-factor solution, however, provided a better fit to the data: TLI = .99 and RMSEA = .04. In the four-factor solution, all items had factor loadings of .31 or higher.

**Confirmatory Factor Analysis (Sample 2).** Using data from Sample 2, we then tested each of the solutions described above (one-, two-, three-, and four-factor) using CFAs. We consulted the results of the EFAs to specify the mapping of items to latent factors and allowed the latent factors to correlate (correlated factors model). In addition, we tested whether there is a global or overarching mindset factor across the items by estimating a series of bifactor models. The bifactor model assumes there is common variance across all items in addition to the items having variance unique to each factor identified in the EFA (Reise, 2012). To ensure the variances of the factors are unique, covariances of the lower-order latent variables are set to be orthogonal to each other in bifactor models. Following the structure of the EFAs, we tested bifactor models using two, three, and four unique (lower-order) factors; each of these models also included a global factor.

Each model was specified using maximum likelihood estimation. We evaluated model fit by consulting RMSEA, TLI, the comparative fit index (CFI), and the standardized root mean square residual (SRMR). Conventional rules of thumb suggest that values of RMSEA less than .06, values of CFI and TLI greater than or equal to .95, and values of SRMR less than or equal to .08 indicate excellent model fit (Hu & Bentler, 1999).

*Correlated factors models*. Table S6 summarizes the results of the confirmatory factor analyses. The one-factor solution (Model A) demonstrated poor model fit even though it allowed the errors of the items that shared a domain and wording to correlate (which none of the other models did): χ²(48) = 214.67, *p* < .001; RMSEA = .18; TLI = .58, CFI = .69; SRMR = .12. The two-factor solution (Model B; six high-ability items, six low-ability items) also demonstrated poor fit: χ²(53) = 164.69, *p* < .001; RMSEA = .14; TLI = .74, CFI = .79; SRMR = .12. The three-factor solution (Model C; six high-ability items, three low-ability instability items, three low-ability malleability items) provided a better fit to the data: χ²(51) = 83.50, *p* = .003; RMSEA = .08; TLI = .92, CFI = .94; SRMR = .06. Finally, the four-factor solution (Model D; three high-ability instability items, three high-ability malleability items, three low-ability instability items, three low-ability malleability items) demonstrated the best fit to the data: χ²(48) = 68.43, *p* = .028; RMSEA = .06; TLI = .95, CFI = .96; SRMR = .05. A formal comparison of the 3- and 4-factor solution using a likelihood ratio test suggested that the 4-factor solution (Model D) represented a better fit to the data than the 3-factor solution (Model C): Δχ²(3) = 15.06, *p* = .002. In the completely standardized (i.e., both latent and observed variables standardized) four-factor solution, all items had loadings between .58 and .91 and were statistically significant (all *p*s < .001).

*Bifactor models*. The bifactor model with two unique factors (Model E; six high-ability items, six low-ability items) demonstrated an adequate fit to the data: χ²(42) = 74.90, *p* = .001; RMSEA = .09; TLI = .90, CFI = .94; SRMR = .05. The bifactor model with three unique factors (Model F; six high-ability items, three low-ability instability items, three low-ability malleability items) did not converge and thus did not produce fit indices. The bifactor model with four unique factors (Model G; three high-ability instability items, three high-ability malleability items, three low-ability instability items, three low-ability malleability items) demonstrated a good fit to the data: χ²(42) = 62.32, *p* = .022; RMSEA = .07; TLI = .94, CFI = .96; SRMR = .05.

A formal comparison of the four-factor correlated factor solution (Model D) with the four-unique-factor bifactor solution (Model G) using a likelihood ratio test revealed no significant difference in the fit of the two solutions: Δχ²(6) = 6.11, *p* = .41. Thus, we chose to retain the simpler, four-factor correlated factors model (Model D). We computed average scale scores for each of the four factors (see Table S7 for zero-order correlations between the four scales for Samples 1 and 2 combined).

**Appendix S2**

**Coding Scheme for Children’s Open-ended Justifications (Study 1)**

The coding scheme used to code children’s explanations was adapted from Heyman and Dweck (1998). Specifically, the content of children’s explanations was coded with respect to whether they mentioned *processes* or *ability*. References to *processes* could include concepts such as effort, practicing, studying, paying attention, working hard, doing math, or receiving help from others. They could also include mention of concepts that implied cognitive change, such as learning or forgetting. References to *ability* could include mention of being smart or “being really good at math,” or mention of cognitive stability, such as remembering or “still knowing” (0 = *ability*, 1 = *processes*). Two coders independently coded children’s responses. The inter-rater reliability was substantial for both questions (κs = .67 and .69, respectively; Landis & Koch, 1977). Responses that were unintelligible or uncodable along these dimensions did not receive a code. The percentages of open-ended justifications that did not receive a code were 34% and 30% for the high- and low-ability vignettes, respectively.

**Appendix S3**

**Measurement Invariance Across Age Groups and Gender (Study 1)**

We assessed the measurement invariance of the GM-C scale using multi-group CFA across two demographic variables: age group (4-, 5-, and 6-year-olds) and gender (girls and boys). If a scale displays measurement invariance across groups, this means that participants from these groups interpret the scale items in the same way—a necessary precondition for any group comparisons (e.g., Lee, 2018). In our case, it is important to establish, for example, that 4-year-olds and 6-year-olds interpret the items in the GM-C in the same way; if they don’t, it would be meaningless to compare these two groups’ mindset scores. We conducted the invariance analyses in a stepwise fashion, starting with a baseline model with no parameters constrained, and then adding parameter constraints in each subsequent step. The baseline model tested for *configural* invariance (i.e., invariance of model form) and thus did not have any parameters constrained. We then constrained item loadings to be invariant to test for *metric* (weak) invariance, and in the last step constrained item loadings and item intercepts to be invariant to test for *scalar* (strong) invariance. To evaluate whether configural invariance was supported, we assessed overall model fit. Configural invariance is supported by either good or adequate model fit. To evaluate whether metric or scalar invariance is supported, we conducted likelihood ratio tests to compare the fit (specifically, Δχ²) between the configural and metric models first and then the metric and scalar models. If model fit did not become substantially worse after imposing a stricter constraint, we concluded that that particular type of invariance is supported.

With respect to age group, the configural model demonstrated adequate fit: χ²(144) = 178.04, *p* = .028; RMSEA = .08; TLI = .92, CFI = .94; SRMR = .09, suggesting invariance in the factor structure of the GM-C across the three age groups. Next, a formal comparison between the configural and metric models indicated that imposing invariant factor loadings did not significantly reduce model fit: Δχ²(16) = 22.98, *p* = .11. Finally, a formal comparison between the metric and scalar models indicated that imposing invariant loadings and intercepts did not significantly reduce model fit: Δχ²(16) = 23.27, *p* = .11. Overall, we find support for configural, metric, and scalar invariance of the GM-C across age groups, suggesting that the items in this scale are interpreted in the same way by children of different ages.

With respect to gender, the configural model demonstrated good fit: χ²(96) = 114.83, *p* = .092; RMSEA = .06; TLI = .95, CFI = .97; SRMR = .07, suggesting invariance in the factor structure of the GM-C between girls and boys. Next, a formal comparison between the configural and metric models indicated that imposing invariant factor loadings did not significantly reduce model fit: Δχ²(8) = 4.38, *p* = .82. Finally, a formal comparison between the metric and scalar models indicated that imposing invariant loadings and intercepts did not significantly reduce model fit: Δχ²(8) = 7.04, *p* = .53. Overall, we find support for configural, metric, and scalar invariance of the GM-C across gender groups, suggesting that the items in this scale are interpreted in the same way by girls and boys (see Tables S8 and S9 for results of the multi-group CFAs).

**Appendix S4**

**Additional Methodological Details of Scale Administration (Study 2)**

Character names were replaced with names from the region (e.g., “This is Iminathi. And here’s something about Iminathi: Iminathi isn’t very good at math. Iminathi gets a lot of math problems wrong on her schoolwork”).^^[[1]](#footnote-1)^^ As in Study 1, for each vignette, children were asked an attention check question (e.g., “Is Iminathi good at math? Or not good at math?”).

*Instability of Low Ability.* The same three instability-of-low-ability items from Study 1 were used here (e.g., “Now here’s a question for you: Will it always be this way? Will Iminathi always be not very good at math? Yes or no?”). One difference from Study 1 concerns the response scale. Because children in this study were older, more response options were offered. Confidence ratings were solicited with the following question: “How sure are you about this? Are you not sure? Sort of sure? Or really sure?” This yielded six possible responses, rather than four (yes *and* really sure = 0, yes *and* sort of sure = .20, yes *and* not sure = .40, no *and* not sure = .60, no *and* sort of sure = .80, no *and* really sure = 1.00). Higher numbers indicated a stronger growth mindset.

*Malleability of Low Ability.* The same three malleability-of-low-ability items from Study 1 were used here (e.g., “Now let me tell you what happened with Iminathi. When Iminathi was a little older, she moved to a school far away. At this school, kids do a lot of math. After Iminathi started at this far-away school, she got to practice math a lot. Iminathi did a lot of math at this school”). Children were then asked whether they thought there was a subsequent change in the character’s abilities (e.g., “Now here’s a question for you: Iminathi was at this school for a long time. When she left this school, how good was she at math? Not at all good? A little not good? A little good? Or very good?”). In addition to offering four response options (rather than two as in Study 1), confidence ratings were solicited with the following question: “How sure are you about this? Are you not sure? Sort of sure? Or really sure?” This yielded a 12-point (rather than six-point) scale, from *not at all good* and *really sure* = 0 to *very good* and *really sure* = 1. Higher numbers indicated a stronger growth mindset. (Results did not change appreciably when we used only children’s first, four-point rating on these items, disregarding their confidence rating [see Appendix S5].)

**Appendix S5**

**Validation Analyses Using a Simplified Malleability Subscale (Study 2)**

In Study 2, the malleability-of-low-ability items involved a two-step question. Children were first asked if they thought there would be a change in the character’s abilities, then confidence ratings were solicited. These two questions yielded a 12-point scale, from *not at all good* and *really sure* = 0 to *very good* and *really sure* = 1.00. Because this level of granularity was a deviation from the GM-C’s original response format (which yielded a six-point scale), we reran all validation analyses using just children’s responses to the first question (*not at all good* = 0, *a little not good* = .33, *a little good* = .67, *very good* = 1). When we did so, the results did not change appreciably.

*Learning Goals.* When both subscale scores were entered into the model simultaneously along with grade level, both the malleability-of-low-ability score (odds ratio = 1.44 [1.17, 1.76], *b* = 0.36 [0.16, 0.57], *SE* = 0.10, *p* < .001) and the instability-of-low-ability score (odds ratio = 1.40 [1.09, 1.81], *b* = 0.34 [0.09, 0.59], *SE* = 0.13, *p* = .009) were significant predictors of children’s goals.

*Challenge-seeking.* When both subscale scores were entered into the model simultaneously along with grade level, children’s score on the instability-of-low-ability subscale was a significant predictor of their puzzle choice (*b* = 0.26 [0.08, 0.45], *SE* = 0.09, *p* = .006), such that children who viewed low ability as less stable were more likely to select a challenging puzzle. Neither children’s score on the malleability-of-low-ability subscale (*p* = .51) nor their grade level (*p* = .30) were significant predictors of their choice.

*Math Achievement.* When both subscale scores were entered into the model simultaneously along with grade level, children’s score on the instability-of-low-ability subscale was a significant predictor of their math achievement (*b* = 2.06 [1.17, 2.95], *SE* = 0.45, *p* < .001), such that children who viewed low ability as less stable earned higher grades in math. Children’s score on the malleability-of-low-ability subscale was not a significant predictor of their math achievement (*p* = .99).

*English Achievement.* When both subscale scores were entered into the model simultaneously along with grade level, children’s score on the instability-of-low-ability subscale was a significant predictor of their English achievement (*b* = 1.63 [0.74, 2.52], *SE* = 0.45, *p* < .001), such that children who viewed low ability as less stable earned higher grades in English. Children’s score on the malleability-of-low-ability subscale was not a significant predictor of their English achievement (*p* = .72).

**References**

Heyman, G. D., & Dweck, C. S. (1998). Children's thinking about traits: Implications for judgments of the self and others. *Child Development*, *69*(2), 391-403. https://doi.org/10.1111/j.1467-8624.1998.tb06197.x

Hu, L. T., & Bentler, P. M. (1995). Evaluating model fit. In R. H. Hoyle (Ed.), *Structural equation modeling: Issues, concepts, and applications* (pp. 76-99). Newbury Park, CA: Sage

Landis, J. R., & Koch, G. G. (1977). The measurement of observer agreement for categorical data. *Biometrics*, 159-174. https://doi.org/10.2307/2529310

Lee, S. T. (2018). Testing for measurement invariance: Does your measure mean the same thing for different participants? *APS Observer*, *31*(8), 32-34.

Reise, S. P. (2012). The rediscovery of bifactor measurement models. *Multivariate Behavioral Research*, *47*(5), 667-696. https://doi.org/10.1080/00273171.2012.715555

Steiger, J. H., & Lind, J. M. (1980, June). *Statistically based tests for the number of common factors.* Paper presented at the annual meeting of the Psychometric Society, Iowa City, IA.

Tucker, L. R., & Lewis, C. (1973). A reliability coefficient for maximum likelihood factor analysis. *Psychometrika*, *38*(1), 1-10.


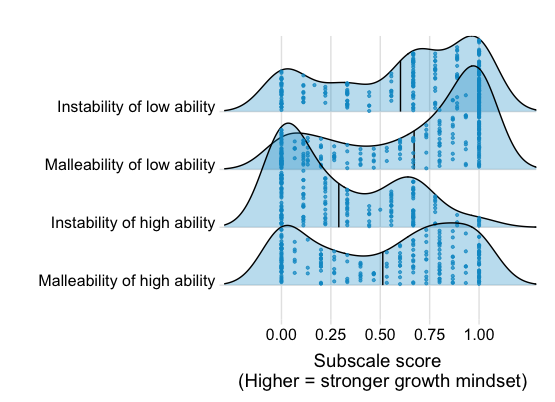


*Figure S1*. Ridgeline plots displaying subscale distributions in Study 1. Points are jittered. Black lines represent means.


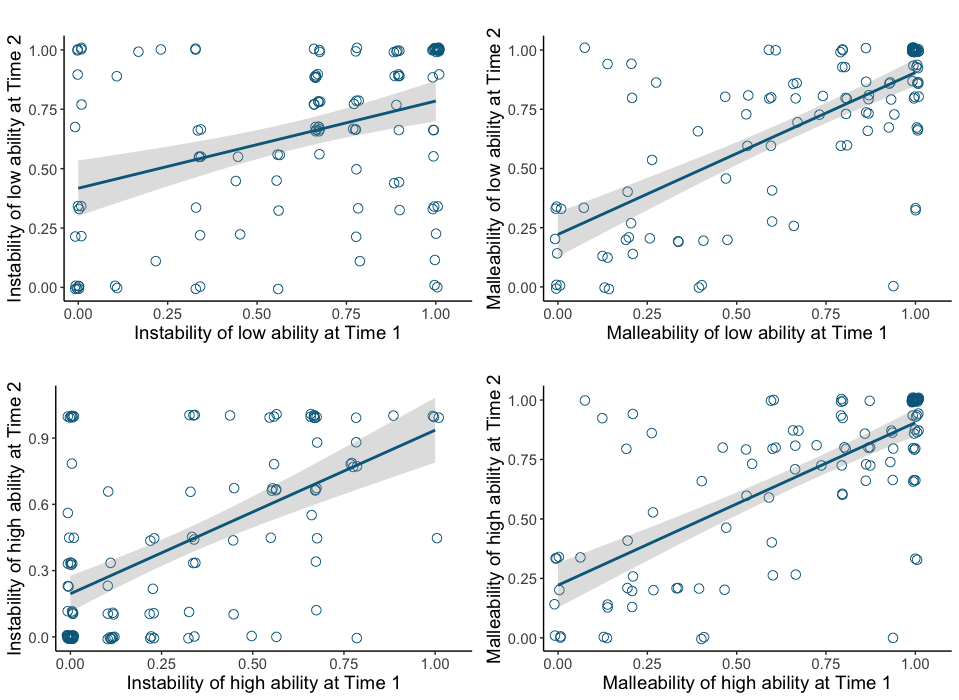


*Figure S2*. Relation between children’s scores on each of the four subscales of the GM-C measure at Time 1 and Time 2 in Study 1. Points are jittered. Band represents ± 1 *SE.*


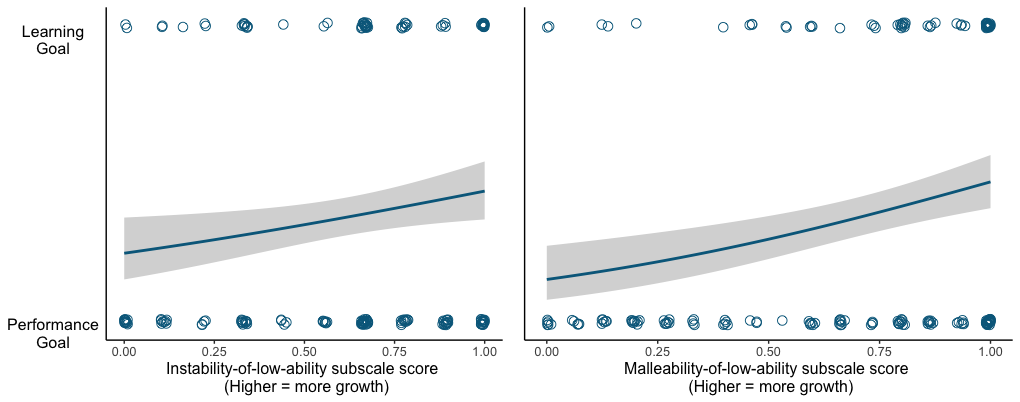


*Figure S3*. Relation between children’s goals and their scores on the instability- and malleability-of-low-ability subscales of the GM-C (Study 1). Points are jittered. Band represents ± 1 *SE.*

**
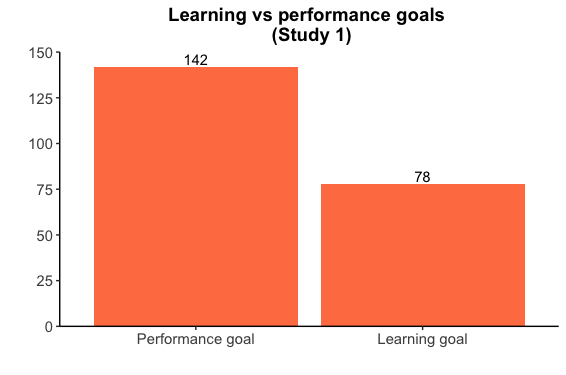

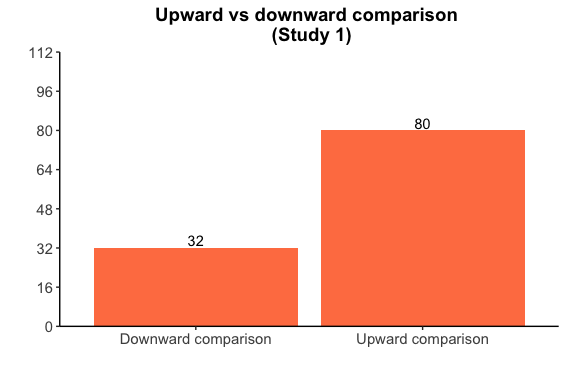

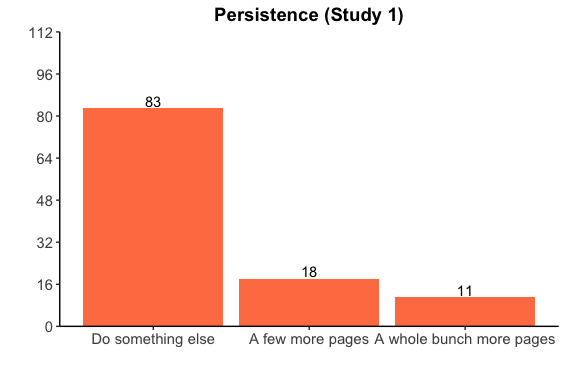
**

*Figure S4.* Distribution of children’s responses for learning goals (Samples 1 and 2), upward versus downward comparisons (Sample 1), and persistence (Sample 1) in Study 1.

**
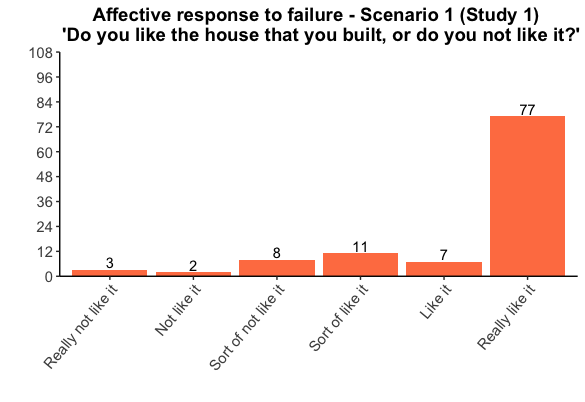

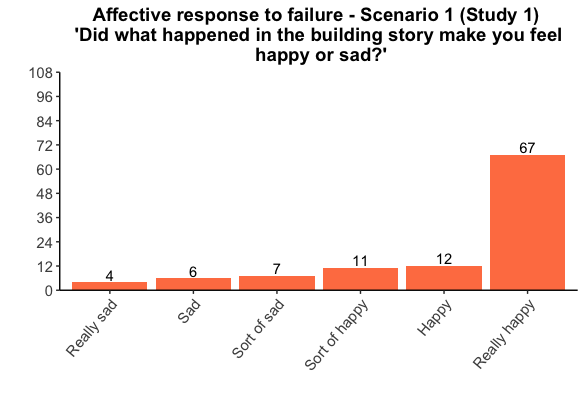
**

**
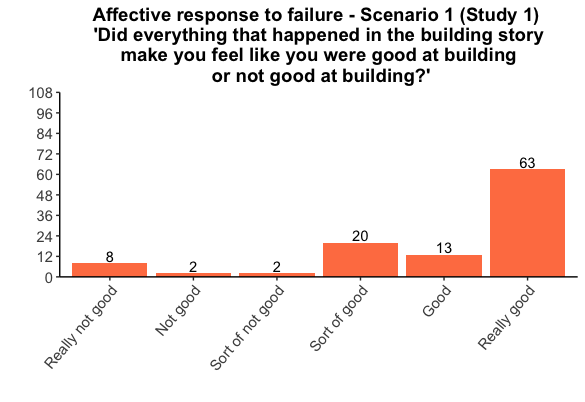
**

*Figure S5.* Distribution of children’s responses for items measuring children’s affective response to failure in Scenario 1 (Study 1).

**
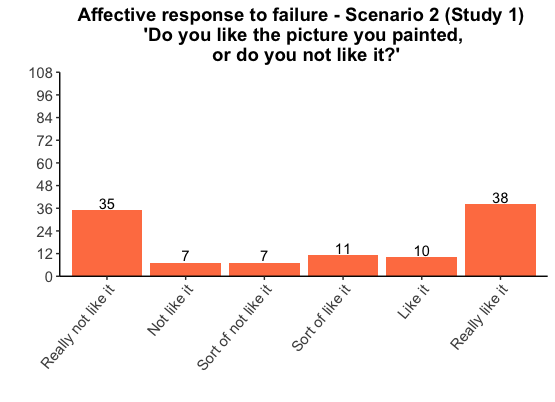

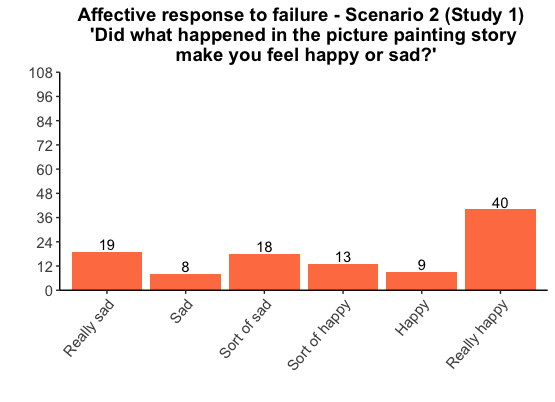

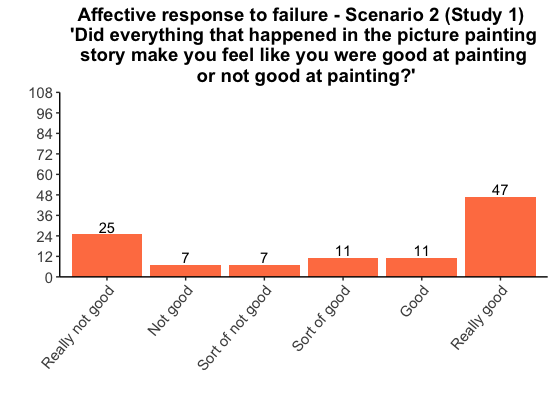
**

*Figure S6.* Distribution of children’s responses for items measuring children’s affective response to failure in Scenario 2 (Study 1).

**
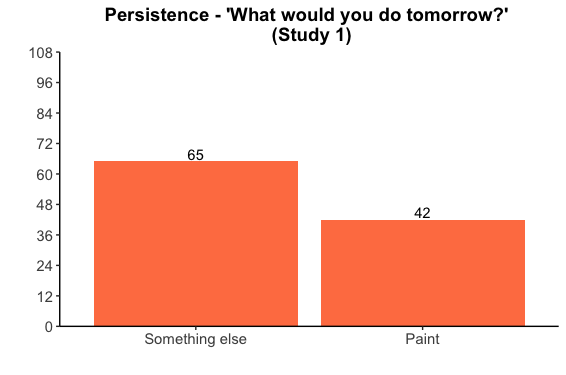

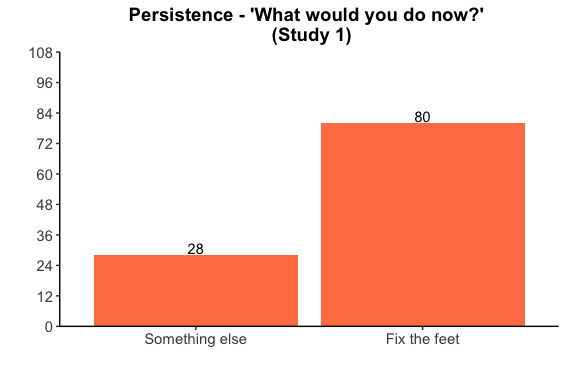
**

*Figure S7.* Distribution of children’s responses for the persistence items (Sample 2 of Study 1).

*
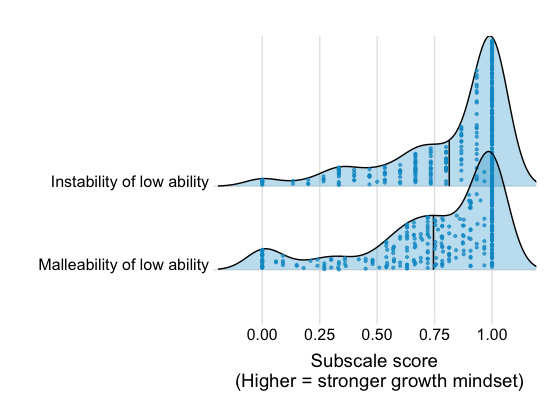
*

*Figure S8*. Ridgeline plots displaying subscale distributions in Study 2. Points are jittered. Black lines represent means.


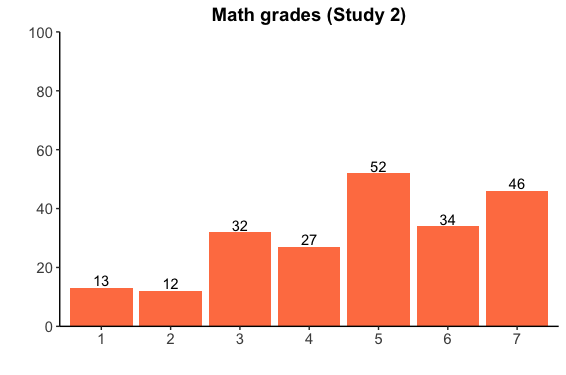

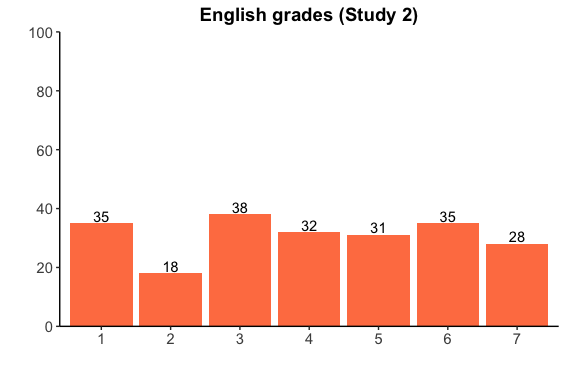

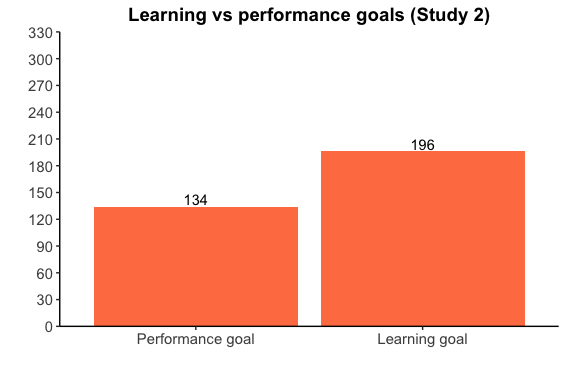

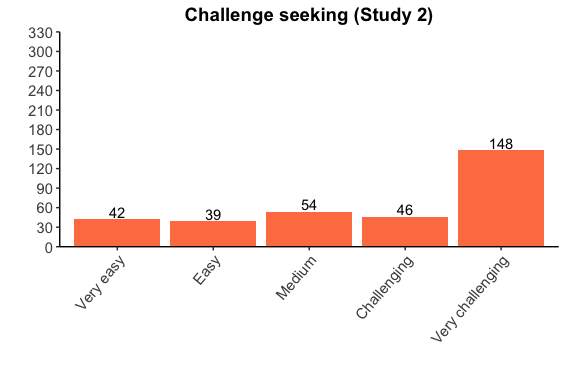


*Figure S9.* Distribution of children’s grades and their responses to items measuring learning goals and challenge seeking (Study 2).


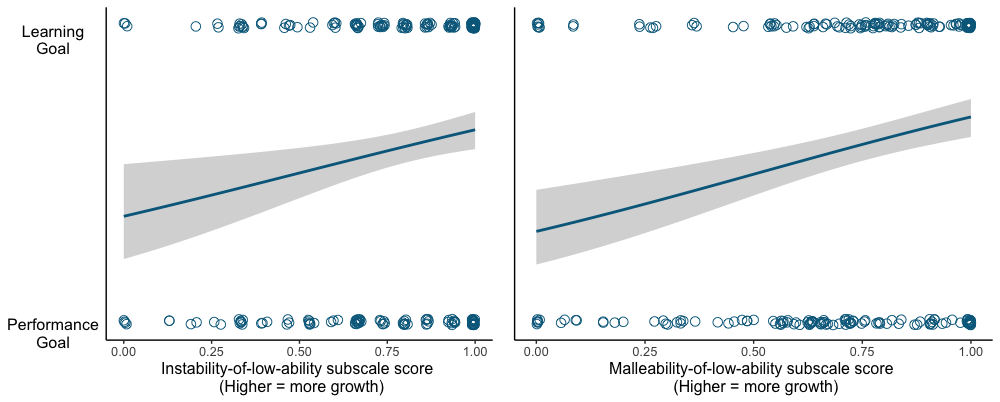


*Figure S10*. Relation between children’s goals and their scores on the instability- and malleability-of-low-ability subscales of the GM-C (Study 2). Points are jittered. Band represents ± 1 *SE.*


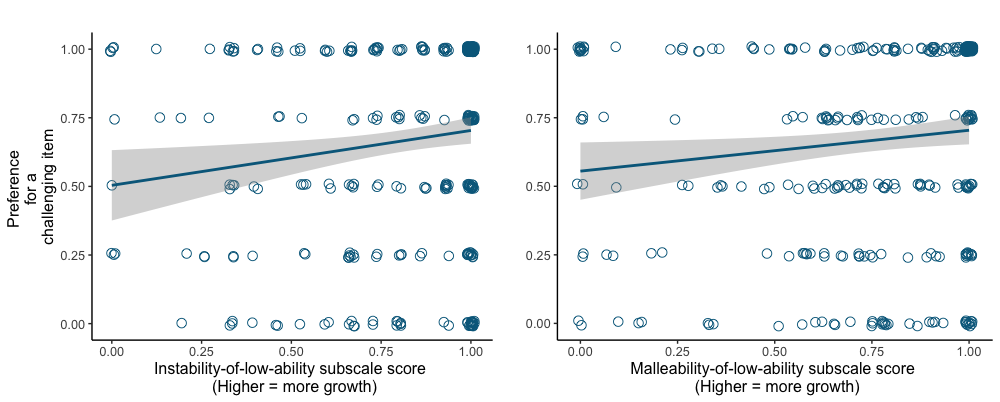


*Figure S11*. Relation between children’s challenge-seeking behavior and their scores on the instability- and malleability-of-low-ability subscales of the GM-C (Study 2). Points are jittered. Band represents ± 1 *SE.*


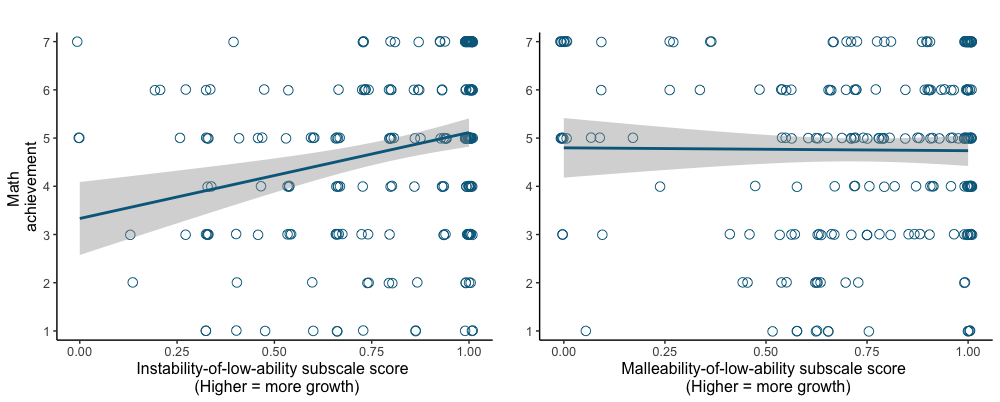


*Figure S12*. Relation between children’s math achievement and their scores on the instability- and malleability-of-low-ability subscales of the GM-C (Study 2). Points are jittered. Band represents ± 1 *SE.*


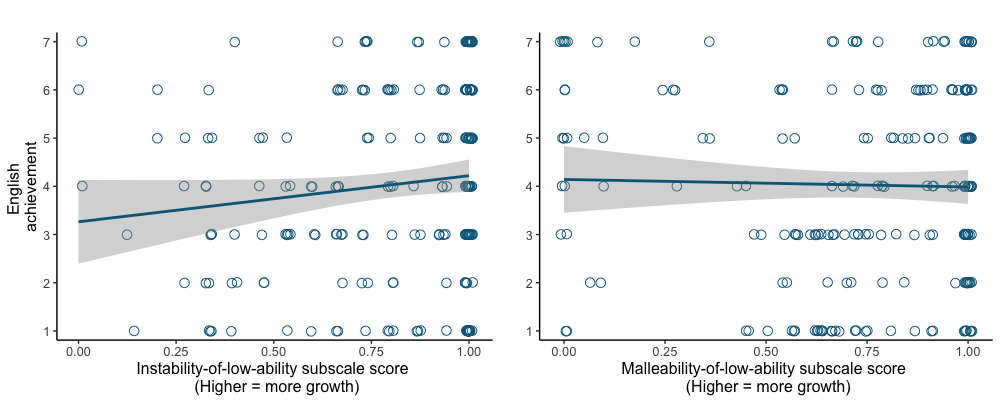


*Figure S13*. Relation between children’s English achievement and their scores on the instability- and malleability-of-low-ability subscales of the GM-C (Study 2). Points are jittered. Band represents ± 1 *SE.*

**Table S1**

*Example Items from GM-C Measure*

| *High-ability vignette (math)*  This is Alex. And here’s something about Alex: Alex is really good at math. Alex gets *all* of the math problems right on her schoolwork.  I just want to make sure you were paying attention: Is Alex good at math? Or not good at math?  Now here’s a question for you: Will it always be this way? Will Alex always be really good at math? [*instability of ability*]  Now let me tell you what happened with Alex. When Alex was a little older, she moved to a school far away. At this school, kids don’t do a lot of math. After Alex started at this far-away school, she didn’t get to practice math very much. Alex didn’t do almost any math at this school.  Now here’s a question for you: Alex was at this school for a long time. When she left this school, was she good at math or not good at math? [*malleability of ability*] |
| --- |
| *Low-ability vignette (math)*  This is Jamie. And here’s something about Jamie: Jamie isn’t very good at math. Jamie gets *a* *lot* of math problems wrong on her schoolwork.  I just want to make sure you were paying attention: Is Jamie good at math? Or not good at math?  Now here’s a question for you: Will it always be this way? Will Jamie always be not very good at math? [*instability of ability*]  Now let me tell you what happened with Jamie. When Jamie was a little older, she moved to a school far away. At this school, kids do a lot of math. After Jamie started at this far-away school, she got to practice math a lot. Jamie did a lot of math at this school.  Now here’s a question for you: Jamie was at this school for a long time. When she left this school, was she good at math or not good at math? [*malleability of ability*] |

*Note*. The math items in GM-C are above. The measure also included items that concerned two other domains: spelling and drawing (see the OSF repository for the full set of items: <https://osf.io/hm2yg/?view_only=7da7b40cd6724fae84f1ed3b493f79b7>).

**Table S2**

*EFA One-Factor Solution Item Loadings and Descriptive Statistics*

| **Items** | F1 | Mean (SD) |
| --- | --- | --- |
| Math (Instability-of-low-ability) | .36 | .56 (.45) |
| Spelling (Instability-of-low-ability) | .29 | .54 (.42) |
| Drawing (Instability-of-low-ability) | .31 | .60 (.43) |
| Math (Malleability-of-low-ability) | .60 | .67 (.40) |
| Spelling (Malleability-of-low-ability) | .55 | .63 (.42) |
| Drawing (Malleability-of-low-ability) | .62 | .63 (.43) |
| Math (Instability-of-high-ability) | .62 | .25 (.38) |
| Spelling (Instability-of-high-ability) | .43 | .24 (.22) |
| Drawing (Instability-of-high-ability) | .63 | .28 (.38) |
| Math (Malleability-of-high-ability) | .86 | .54 (.41) |
| Spelling (Malleability-of-high-ability) | .83 | .50 (.42) |
| Drawing (Malleability-of-high-ability) | .85 | .49 (.43) |

*Note.* Factors were extracted using maximum likelihood and rotated using the oblimin method. Loadings <|.20| are not displayed.

**Table S3**

*EFA Two-Factor Solution Item Loadings and Descriptive Statistics*

| **Items** | F1  (Low ability) | F2  (High ability) | Mean (SD) |
| --- | --- | --- | --- |
| Math (Instability-of-low-ability) | .21 |  | .56 (.45) |
| Spelling (Instability-of-low-ability) | .38 |  | .54 (.42) |
| Drawing (Instability-of-low-ability) | .22 |  | .60 (.43) |
| Math (Malleability-of-low-ability) | .88 |  | .67 (.40) |
| Spelling (Malleability-of-low-ability) | .92 |  | .63 (.42) |
| Drawing (Malleability-of-low-ability) | .85 |  | .63 (.43) |
| Math (Instability-of-high-ability) |  | .48 | .25 (.38) |
| Spelling (Instability-of-high-ability) |  | .30 | .24 (.22) |
| Drawing (Instability-of-high-ability) |  | .50 | .28 (.38) |
| Math (Malleability-of-high-ability) |  | .84 | .54 (.41) |
| Spelling (Malleability-of-high-ability) |  | .93 | .50 (.42) |
| Drawing (Malleability-of-high-ability) |  | .90 | .49 (.43) |

*Note.* Factors were extracted using maximum likelihood and rotated using the oblimin method. Loadings <|.20| are not displayed.

**Table S4**

*EFA Three-Factor Solution Item Loadings and Descriptive Statistics*

| **Items** | F1  (Instability-of-low-ability) | F2  (Malleability-of-low-ability) | F3  (High ability) | Mean (SD) |
| --- | --- | --- | --- | --- |
| Math (Instability-of-low-ability) | .77 |  |  | .56 (.45) |
| Spelling (Instability-of-low-ability) | .71 |  |  | .54 (.42) |
| Drawing (Instability-of-low-ability) | .92 |  |  | .60 (.43) |
| Math (Malleability-of-low-ability) |  | .85 |  | .67 (.40) |
| Spelling (Malleability-of-low-ability) |  | .95 |  | .63 (.42) |
| Drawing (Malleability-of-low-ability) |  | .85 |  | .63 (.43) |
| Math (Instability-of-high-ability) | .35 |  | .45 | .25 (.38) |
| Spelling (Instability-of-high-ability) |  |  | .27 | .24 (.22) |
| Drawing (Instability-of-high-ability) | .38 |  | .46 | .28 (.38) |
| Math (Malleability-of-high-ability) |  |  | .84 | .54 (.41) |
| Spelling (Malleability-of-high-ability) |  |  | .93 | .50 (.42) |
| Drawing (Malleability-of-high-ability) |  |  | .89 | .49 (.43) |

*Note.* Factors were extracted using maximum likelihood and rotated using the oblimin method. Loadings <|.20| are not displayed.

**Table S5**

*EFA Four-Factor Solution Item Loadings and Descriptive Statistics*

| **Items** | F1  (Instability-of-low-ability) | F2  (Malleability-of-low-ability) | F3  (Instability-of-high-ability) | F4  (Malleability-of-high-ability) | Mean (SD) |
| --- | --- | --- | --- | --- | --- |
| Math (Instability-of-low-ability) | .83 |  |  |  | .56 (.45) |
| Spelling (Instability-of-low-ability) | .74 |  |  |  | .54 (.42) |
| Drawing (Instability-of-low-ability) | .82 |  |  |  | .60 (.43) |
| Math (Malleability-of-low-ability) |  | .85 |  |  | .67 (.40) |
| Spelling (Malleability-of-low-ability) |  | .94 |  |  | .63 (.42) |
| Drawing (Malleability-of-low-ability) |  | .84 |  |  | .63 (.43) |
| Math (Instability-of-high-ability) |  |  | .66 |  | .25 (.38) |
| Spelling (Instability-of-high-ability) |  |  | .31 |  | .24 (.22) |
| Drawing (Instability-of-high-ability) |  |  | .76 |  | .28 (.38) |
| Math (Malleability-of-high-ability) |  |  |  | .78 | .54 (.41) |
| Spelling (Malleability-of-high-ability) |  |  |  | .84 | .50 (.42) |
| Drawing (Malleability-of-high-ability) |  |  |  | .95 | .49 (.43) |

*Note.* Factors were extracted using maximum likelihood and rotated using the oblimin method. Loadings <|.20| are not displayed.

**Table S6**

*Fit Indices from the Confirmatory Factor Analyses*

|  |  | | | Fit Indices | | | | |
| --- | --- | --- | --- | --- | --- | --- | --- | --- |
| Model | Number of factors | χ^2^ | df | | RMSEA | CFI | TLI | SRMR |
| A | 1 | 214.67 | 48 | | .18 | .69 | .58 | .12 |
| Correlated Factors Models | | | | | | | | |
| B | 2 | 164.69 | 53 | | .14 | .79 | .74 | .12 |
| C | 3 | 83.50 | 51 | | .08 | .94 | .92 | .06 |
| **D** | **4** | **68.43** | **48** | | **.06** | **.96** | **.95** | **.05** |
| Bifactor Models | | | | | | | | |
| E | 1 global, 2 unique | 74.90 | 42 | | .09 | .94 | .90 | .05 |
| F | 1 global, 3 unique | — | — | | — | — | — | — |
| G | 1 global, 4 unique | 62.32 | 42 | | .07 | .96 | .94 | .05 |

*Note*. RMSEA = Root Mean Square Error of Approximation; CFI = Comparative Fit Index; TLI = Tucker-Lewis Index; SRMR = Standardized Root Mean Square Residual. Model F did not converge to a solution. The bolded model (Model D) is the model that was ultimately retained.

**Table S7**

*Means, SDs, and Correlations Among the Four Subscales of the GM-C Scale in Study 1 (Samples 1 and 2 combined)*

|  | *M* (*SD*) | 1. | 2. | 3. | 4. |
| --- | --- | --- | --- | --- | --- |
| 1. Instability of low ability | .60 (0.36) | — |  |  |  |
| 2. Malleability of low ability | .67 (0.36) | .29*** | — |  |  |
| 3. Instability of high ability | .29 (0.31) | .34*** | .41*** | — |  |
| 4. Malleability of high ability | .51 (0.37) | .22*** | .46*** | .58*** | — |

*Note.* Possible range = [0, 1]. Observed range = [0, 1]. Higher numbers reflect stronger growth mindsets. Instability concerned the possibility of change over time; malleability concerned the possibility of change via intervention (i.e., moving to a different school). *** *p* < .001.

**Table S8**

*Testing for Measurement Invariance Across Age Groups in Study 1*

| Model | χ^2^ | df | Δχ² | Δdf | Δ*p* | RMSEA | CFI |
| --- | --- | --- | --- | --- | --- | --- | --- |
| Configural invariance (model form) | 178.04 | 144 |  |  |  | .082 | .94 |
| Metric invariance (loadings) | 201.02 | 160 | 22.98 | 16 | .015 | .086 | .93 |
| Scalar invariance (loadings and intercepts) | 224.29 | 176 | 23.27 | 16 | .007 | .089 | .92 |

**Table S9**

*Testing for Measurement Invariance Across Gender Groups in Study 1*

| Model | χ^2^ | df | Δχ² | Δdf | Δ*p* | RMSEA | CFI |
| --- | --- | --- | --- | --- | --- | --- | --- |
| Configural invariance (model form) | 114.83 | 96 |  |  |  | .061 | .97 |
| Metric invariance (loadings) | 119.21 | 104 | 4.38 | 8 | .054 | .053 | .97 |
| Scalar invariance (loadings and intercepts) | 126.25 | 112 | 7.04 | 8 | .023 | .049 | .97 |

**Table S10**

*Cronbach’s Alphas (and 95% CIs), Overall and by Age, for the Four Subscales of the GM-C Scale in Study 1 (Samples 1 and 2 combined)*

|  | Overall  (*N* = 220) | 4-year-olds  (*n* = 74) | 5-year-olds  (*n* = 73) | 6-year-olds  (*n* = 73) |
| --- | --- | --- | --- | --- |
| 1. Instability of low ability | .82 [.78, .86] | .82 [.75, .89] | .83 [.76, .90] | .83 [.76, .90] |
| 2. Malleability of low ability | .90 [.88, .92] | .87 [.82, .92] | .92 [.89, .95] | .88 [.83, .93] |
| 3. Instability of high ability | .71 [.64, .77] | .63 [.48, .77] | .66 [.53, .80] | .80 [.72, .88] |
| 4. Malleability of high ability | .89 [.86, .92] | .89 [.84, .93] | .89 [.85, .93] | .89 [.84, .93] |

*Note.* None of the differences between ages in alpha values within a subscale were statistically significant (*p*s > .12).

**Table S11**

*Examples of Open-Ended Justifications in Study 1*

| Code | Justification |
| --- | --- |
| Processes | “Because she practiced a lot of things”  “Because she learned so much math when she got home, she told her mom how good she was at math”  “Because she was learning about it”  “’Cause they had math there and then they got better and better” |
| Ability | “Because she is not good at writing numbers”  “Because she doesn’t know math and she will always be like that”  “Because she was really good at another school”  “Because she’s good at math and when she moves to that school she’s good at math at that school too” |

**Table S12**

*Results of the ordinal regression model predicting children’s persistence (*1 = *do a whole bunch more pages,* 0.5 *= do a few more pages,* 0 *= do something else) from their scores on the instability-of-low-ability and malleability-of-low-ability subscales and age in Study 1.*

|  | *b* | *SE* | *p* | 95% *CI* | |
| --- | --- | --- | --- | --- | --- |
|  |  |  |  |  |  |
| Instability of low ability | −0.73 | 0.62 | .24 | −1.97 | 0.48 |
| Malleability of low ability | 0.71 | 0.68 | .30 | −0.58 | 2.10 |
| Age | 0.26 | 0.28 | .35 | −0.29 | 0.83 |
|  |  |  |  |  |  |

**Table S13**

*Results of the logistic regression model predicting children’s upward comparison (*1 = *upward comparison,* 0 = *downward comparison) from their scores on the instability-of-low-ability and malleability-of-low-ability subscales and age in Study 1. Coefficients are in log odds.*

|  | *b* | *SE* | *p* | 95% *CI* | |
| --- | --- | --- | --- | --- | --- |
|  |  |  |  |  |  |
| Intercept | 2.24 | 1.50 | .14 | −0.66 | 5.26 |
| Instability of low ability | −1.01 | 0.62 | .10 | −2.28 | 0.17 |
| Malleability of low ability | 0.93 | 0.63 | .14 | −0.30 | 2.20 |
| Age | −0.24 | 0.28 | .38 | −0.79 | 0.30 |
|  |  |  |  |  |  |

**Table S14**

*Results of the regression model predicting children’s affective response to failure (higher = more positive reaction) from their scores on the instability-of-low-ability and malleability-of-low-ability subscales and age in Study 1.*

|  | *b* | *SE* | *p* | 95% *CI* | |
| --- | --- | --- | --- | --- | --- |
|  |  |  |  |  |  |
| **Intercept** | **1.21** | **0.16** | **<.001** | **0.90** | **1.52** |
| Instability of low ability | 0.02 | 0.06 | .75 | −0.11 | 0.15 |
| Malleability of low ability | 0.02 | 0.07 | .76 | −0.11 | 0.15 |
| **Age** | **−0.10** | **0.03** | **<.001** | −**0.15** | **−0.04** |
|  |  |  |  |  |  |

*Note*. Bolded coefficients are statistically significant.

**Table S15**

*Results of the regression model predicting children’s persistence on the second measure (higher = more persistence) from their scores on the instability-of-low-ability and malleability-of-low-ability subscales and age in Study 1.*

|  | *b* | *SE* | *p* | 95% *CI* | |
| --- | --- | --- | --- | --- | --- |
|  |  |  |  |  |  |
| Intercept | 0.17 | 0.27 | .52 | −0.35 | 0.70 |
| Instability of low ability | −0.003 | 0.11 | .98 | −0.22 | 0.21 |
| Malleability of low ability | 0.03 | 0.11 | .81 | −0.20 | 0.25 |
| Age | 0.07 | 0.05 | .15 | −0.03 | 0.16 |
|  |  |  |  |  |  |

**Table S16**

*Results of the logistic regression model predicting children’s goals (1 = learning goals, 0 = performance goals) from their scores on the instability-of-high-ability and malleability-of-high-ability subscales and age in Study 1. Coefficients are in log odds.*

|  | *b* | *SE* | *p* | 95% *CI* | |
| --- | --- | --- | --- | --- | --- |
|  |  |  |  |  |  |
| **Intercept** | **−4.62** | **1.07** | **<.001** | **−6.78** | **−2.57** |
| Instability of high ability | 0.34 | 0.58 | .56 | −0.79 | 1.47 |
| Malleability of high ability | 0.47 | 0.48 | .33 | −0.48 | 1.43 |
| **Age** | **0.66** | **0.19** | **<.001** | **0.30** | **1.03** |
|  |  |  |  |  |  |

*Note*. Bolded coefficients are statistically significant.

**Table S17**

*Results of the ordinal regression model predicting children’s persistence (1 = do a whole bunch more pages, 0.5 = do a few more pages, 0 = do something else) from their scores on the instability-of-high-ability and malleability-of-high-ability subscales and age in Study 1.*

|  | *b* | *SE* | *p* | 95% *CI* | |
| --- | --- | --- | --- | --- | --- |
|  |  |  |  |  |  |
| Instability of high ability | 0.09 | 0.92 | .93 | −1.73 | 1.93 |
| Malleability of high ability | −0.18 | 0.71 | .80 | −1.62 | 1.20 |
| Age | 0.39 | 0.27 | .15 | −0.13 | 0.93 |
|  |  |  |  |  |  |

**Table S18**

*Results of the logistic regression model predicting children’s upward comparison (1 = upward comparison, 0 = downward comparison) from their scores on the instability-of-high-ability and malleability-of-high-ability subscales and age in Study 1. Coefficients are in log odds.*

|  | *b* | *SE* | *p* | 95% *CI* | |
| --- | --- | --- | --- | --- | --- |
|  |  |  |  |  |  |
| Intercept | 1.36 | 1.41 | .33 | −1.38 | 4.18 |
| Instability of high ability | 0.30 | 0.88 | .73 | −1.43 | 2.05 |
| Malleability of high ability | −0.38 | 0.66 | .56 | −1.69 | 0.94 |
| Age | −0.06 | 0.26 | .82 | −0.57 | 0.45 |
|  |  |  |  |  |  |

**Table S19**

*Results of the regression model predicting children’s affective response to failure (higher = more positive reaction) from their scores on the instability-of-high-ability and malleability-of-high-ability subscales and age in Study 1.*

|  | *b* | *SE* | *p* | 95% *CI* | |
| --- | --- | --- | --- | --- | --- |
|  |  |  |  |  |  |
| **Intercept** | **1.26** | **0.15** | **<.001** | **0.96** | **1.56** |
| Instability of high ability | −0.02 | 0.08 | .84 | −0.18 | 0.14 |
| Malleability of high ability | −0.07 | 0.07 | .36 | −0.21 | 0.08 |
| **Age** | **−0.09** | **0.03** | **<.001** | **−0.15** | **−0.04** |
|  |  |  |  |  |  |

*Note*. Bolded coefficients are statistically significant.

**Table S20**

*Results of the regression model predicting children’s persistence on the second measure (higher = more persistence) from their scores on the instability-of-high-ability and malleability-of-high-ability subscales and age in Study 1.*

|  | *b* | *SE* | *p* | 95% *CI* | |
| --- | --- | --- | --- | --- | --- |
|  |  |  |  |  |  |
| Intercept | 0.13 | 0.26 | .62 | −0.38 | 0.64 |
| Instability of high ability | −0.10 | 0.14 | .48 | −0.37 | 0.18 |
| Malleability of high ability | 0.14 | 0.12 | .27 | −0.11 | 0.38 |
| Age | 0.07 | 0.05 | .11 | −0.02 | 0.16 |
|  |  |  |  |  |  |

**Table S21**

*Cronbach’s Alphas (and 95% CIs), Overall and by Grade, for the Two Subscales of the GM-C Scale in Study 2*

|  | Overall | 4^th^ graders | 5^th^ graders |
| --- | --- | --- | --- |
| 1. Instability of low ability | .70 [.65, .76] | .63 [.51, .76] | .65 [.53, .76] |
| 2. Malleability of low ability | .90 [.88, .92] | .91 [.87, .94] | .89 [.85, .93] |

*Note.* None of the differences between grades in alpha values within a subscale were statistically significant (*p*s > .48). We did not calculate alphas for children in Grades 2 and 3 separately because the sample sizes for each of these grades were too small.

1. The scale was also translated to Afrikaans and Xhosa so that adult facilitators and children had access to both English and translated versions of the scale. [↑](#footnote-ref-1)
